# Deep Reinforcement Learning Based Optical and Acoustic Dual Channel Multiple Access in Heterogeneous Underwater Sensor Networks

**DOI:** 10.3390/s22041628

**Published:** 2022-02-18

**Authors:** Enhong Liu, Rongxi He, Xiaojing Chen, Cunqian Yu

**Affiliations:** 1 College of Information Science and Technology, Dalian Maritime University, Dalian 116026, China; leh@dlmu.edu.cn (E.L.); chenxiaojing@dlmu.edu.cn (X.C.); yucunqian@dlmu.edu.cn (C.Y.); 2 School of Electrical Engineering, Dalian University of Science and Technology, Dalian 116052, China

**Keywords:** Media Access Control (MAC) protocol, hybrid optical-acoustic underwater sensor networks, heterogeneous networks, deep reinforcement learning

## Abstract

In this paper, we investigate how to efficiently utilize channel bandwidth in heterogeneous hybrid optical and acoustic underwater sensor networks, where sensor nodes adopt different Media Access Control (MAC) protocols to transmit data packets to a common relay node on optical or acoustic channels. We propose a new MAC protocol based on deep reinforcement learning (DRL), referred to as optical and acoustic dual-channel deep-reinforcement learning multiple access (OA-DLMA), in which the sensor nodes utilizing the OA-DLMA protocol are called agents, and the remainder are non-agents. The agents can learn the transmission patterns of coexisting non-agents and find an optimal channel access strategy without any prior information. Moreover, in order to further enhance network performance, we develop a differentiated reward policy that rewards specific actions over optical and acoustic channels differently, with priority compensation being given to the optical channel to achieve greater data transmission. Furthermore, we have derived the optimal short-term sum throughput and channel utilization analytically and conducted extensive simulations to evaluate the OA-DLMA protocol. Simulation results show that our protocol performs with near-optimal performance and significantly outperforms other existing protocols in terms of short-term sum throughput and channel utilization.

## 1. Introduction

As we all know, over 70% of the Earth’s surface is covered by the ocean [1]. With the ever-greater exploitation of terrestrial resources, there is also an increasing demand for new technologies to develop marine resources. With an extensive range of applications including aided navigation [2], unmanned exploration [3], or surveillance [4], seismic reconnaissance [5], and so forth, Underwater Sensor Networks (UWSNs) have been considered as one of the most promising approaches for efficiently exploring and observing the ocean [6].

A stable, reliable, and effective marine development technology is regarded as essential to explore submarine resources. Due to the high attenuation of the electromagnetic wave in water, the signal attenuation will be aggravated with the increase of frequency [7]. Although optical communication has an overwhelming advantage in speed, power consumption, volume, and other aspects, its short communication distance restricts further development [8]. The UWSN that relies on acoustic technologies, referred to as Underwater Acoustic Sensor Network (UASN), has dominated underwater communication for decades since the acoustic signal can provide relatively long-distance and stable communication [9]. Contrarily, the UASN is generally accompanied by inherent narrow bandwidth [10] and non-negligible long propagation delay caused by the slow acoustic speed of 1500 m/s, resulting in low network throughput and channel utilization. Therefore, it is critical and urgent for UASNs to improve the network throughput and channel utilization [11].

Media Access Control (MAC) protocol is one of the crucial technologies in the UWSNs for sensor nodes to share the common underwater channels [12]. Since the MAC protocol can ultimately affect the performance of the underwater networks, it is fundamental and significant to conceive a reliable MAC protocol [13] for achieving high performance. Compared to the mature terrestrial wireless communication system, the UWSN is under exploration and not systematic yet [14]. Due to the harsh underwater environment, the terrestrial MAC protocols cannot be directly applied in the UWSNs. Over the past decades, various specific MAC protocols have been proposed for UWSNs to elevate network performance. Especially in recent years, numerous studies [8,15,16] have focused on the hybrid MAC protocols to achieve a higher performance for Hybrid Optical and Acoustic UWSNs (HOA-UWSNs) that combine the advantages of high-speed optical transmission and reliable long-range acoustic transmission. In the HOA-UWSNs, bulk data streaming and instant messages tend to pass through the optical channel, and acoustic communication carries short packages and non-instant messages [15]. Nodes equipped with optical and acoustic transceivers can appropriately choose the available transmission mode according to the Signal to Noise Ratio (SNR) value and channel conditions [8]. In fact, in the HOA-UWSNs, the optical channel and acoustic channel can be treated as two disparate channels with no interference.

Though the improved optical and acoustic MAC protocols indeed have promoted the network performance, a common precondition of the series is that the global environmental information (the propagation delay, the transmission methods, including channel reservation, data forwarding, etc.) is supposed to be known among nodes [8,16]. In other words, nodes should acquire the corresponding information through some operations, such as control beacon exchanges, or the information is stored in the data message structure [16]. Undoubtedly, the underwater MAC design is severely burdened with those related preliminary operations.

Innovatively, the rise of intelligent algorithms provides a new method to cope with the demand for preliminary information, while meanwhile network performance improvement can also be achieved. The principal reason is that the intelligent algorithms are capable of interacting with the environment and choosing the appropriate actions according to the established objectives via learning and training [17]. A few works [18,19,20] have already brought Deep Reinforcement Learning (DRL) algorithm or Q-learning in the MAC design. Although enhanced network performance without global information can be fulfilled, the mentioned protocols merely concentrated the homogeneous systems where all nodes employ the same MAC protocol. The heterogeneous architecture in which nodes use diverse MAC protocols assists network models and applications in approaching the real world [21].

It has been demonstrated that Deep Q-Network (DQN) [22] can be adopted into heterogeneous underwater MAC protocols [19,23,24] as well to approach the complexity of the real world and meanwhile elevate the network performance. However, the foregoing DRL-based MAC protocols only roughly adapt to the heterogeneous underwater acoustic networks [23,24]. Due to the complex networking and inherent different transmission characteristics of underwater optical and acoustic communication, these existing DRL-based acoustic protocols are not feasible for underwater hybrid optical and acoustic networks. Meanwhile, as far as we know, there is little research concentrating on the heterogeneous MAC protocol fusing optical and acoustic modes, which can make full use of the advantages of optical and acoustic transmissions.

Motivated by the aforementioned considerations, in this paper we propose a new MAC protocol for heterogeneous hybrid optical and acoustic underwater sensor networks, referred to as optical and acoustic dual-channel deep-reinforcement learning multiple access (OA-DLMA). In the HOA-UWSN, the underwater sensor nodes adopting the OA-DLMA protocol are named agents, and the remaining nodes are non-agents that employ other slotted MAC protocols. Our protocol enables the agent nodes equipped with optical-acoustic transceivers to adaptively transmit data packets by optical or acoustic channel towards the relay node. The agents can interact with the environment and learn the optimal transmission strategy when coexisting with non-agent nodes from a series of observations and actions to achieve the goal of performance optimization. In this way, the agents can make full use of the available time slots on both the acoustic and optical channels. Ultimately, the average channel utilization and short-term sum throughput will be enhanced.

The main contributions of our work can be summarized as follows.

- To improve the network performance of UWSNs, we construct a heterogeneous underwater sensor network framework that consists of hybrid optical and acoustic substructures. In the hybrid framework, source nodes can fulfill information interaction with the relay node via optical or acoustic channels. The two kinds of channels are jointly liable for their respective transmissions. Namely, the transmissions on each type of channel will not interfere with each other. As a result, the advantages of rapid transmission and high bandwidth of the optical mode and the stable and long-range transmission of the acoustic mode will be realized effectively.
- For the first time, we introduce the DRL technique into hybrid optical and acoustic dual-channel MAC design and propose a DRL-based MAC protocol for the constructed HOA-UWSN model, referred to as OA-DLMA, where a node applying the OA-DLMA protocol is regarded as an agent and the agent can learn to find an optimal access policy without preliminary knowledge of non-agent nodes. Consequently, the agent nodes can be trained through an effective training mechanism to capture and utilize the underutilized channels that are not entirely consumed by other nodes. It is revealed that the OA-DLMA protocol performs well even without additional prior information or handshake mechanism.
- To further improve the network performance, priority compensation for the optical channel is encouraged since the optical channel possess more data transmission capability. We set a distinguishing reward policy to differentiate the feedback of specific actions on optical and acoustic channels. Specifically, successful optical transmissions will gain larger rewards, while successful acoustic transmissions will obtain smaller rewards.

The rest of this paper is organized as follows. In Section 2, we comprehensively review the related work. The fundamental knowledge of Q-learning and Deep Q-learning algorithms are explained in Section 3. In Section 4, we introduce the system model of the overall network. We describe the details of the OA-DLMA protocol in Section 5. In Section 6, we give the performance analysis and simulation results in different heterogeneous environments. Finally, we conclude the paper in Section 7.

## 2. Related Work

As a promising technique to explore and observe the ocean, UWSNs have drawn great attention from academia, industry, and governments over the past few years. Underwater applications can be implemented through three typical communication techniques, including acoustic, optical and radio frequency (RF) methods. It is widely known that the hydroacoustic technique supports long-range communication among nodes, optical wave guarantees high-speed transmission, while RF technique is seldom deployed in the underwater environment since it suffers from serious attenuation and requires huge antennas [9].

Associated with a particular transmission technique, MAC protocol design is vital for UWSNs to effectively share the channel among diverse underwater nodes [12,25]. Generally, underwater acoustic MAC protocol has dominated the underwater MAC for decades, which are roughly divided into two categories: contention-based and contention-free [26]. Contention-free protocols allocate channel resources in a predefined way. This group minimizes the conflicts at the cost of additional constraints [27], such as frequency division multiple access (FDMA), time division multiple access (TDMA), and code division multiple access (CDMA) [28]. Those approaches stipulate that only one node can access the channel in a regulated fixed segment, and no interference occurs among nodes. More efforts have focused on the contention-based protocols of UWSNs, which access the channels more dynamically and flexibly. Huge amounts of contention-based protocols have thus been proposed, mainly including two classifications of random-access and handshake manner. As a typical random-access protocol, ALOHA protocol [29] permits a node to simply start its transmission whenever it has data ready for delivery. Slotted-ALOHA (S-ALOHA) [30] is considered as a variant ALOHA protocol to address the frequent retransmission and collisions caused by random behaviors of ALOHA. S-ALOHA has to send packets at the beginning of each time slot and shares the same time synchronization in order to reduce the collision, the backoff mechanism is employed at the same time. However, S-ALOHA may exhibit the same deficient performance as ALOHA because of the presence of very high delays [31]. With regard to the more prevalent handshake-based protocols that concentrate on capturing the channel prior to message sending, Molins et al. proposed the slotted floor acquisition multiple access (Slotted-FAMA) protocol [32] that uses a four-way handshake mechanism (RTS/CTS/DATA/CTS) with slotted time to mitigate collisions. However, Slotted-FAMA brings more propagation delay and multi-RTS attempt problems. As a semblable Slotted-FAMA protocol, the T-lohi protocol [33] sends a short frame ahead to compete for the channel, whereas only one node can transmit in the channel at the current time.

As an attractive and feasible alternative, optical wireless communication has recently attracted great interest. Optical signals support higher data rates at a low latency level compared to acoustic counterparts because of its higher bandwidth [34]. Meanwhile, seawater presents a reduced absorption window with wavelengths from 450 nm to 550 nm, which corresponds to the blue–green light [35]. Unfortunately, the performance of optical UWSNs is currently limited to short range [36]. As a result, a new trend that integrates the two technologies has arisen, where optical technique demonstrates efficiency to compensate for the shortcomings of acoustic transmission, and acoustic technique can also behave as a substitute for optical mode to finish long-range transmission.

As early as 2010, researchers in Reference [37] started to develop an integrated underwater optical acoustic communication system which complements and integrates with existing acoustic systems. The hybrid mode contributes to offer high data rates and low latency when within optical range, combined with long range and robustness of acoustics when outside of optical range. The authors of Reference [38] also point out the common trends that allow underwater devices to incorporate different physical communication technologies. The supplementary technology shows the ability to compensate for existing shortcomings through its advantages. In other words, the optical signal usually carries the high-speed data information, and the acoustic signal is loaded with low-bandwidth assistant control information and maintains the data transmission once the optical link fails [16].

The authors in [8] put forward a novel Optical-Acoustic hybrid Underwater Wireless Sensor Network (OA-UWSN), which exactly provides the preliminary knowledge of hybrid transmissions. The design employs optical and acoustic communications for high-speed transmission at close range and transmitting control commands and node localization, respectively. Therewith, the authors in [39] formally propose a novel energy-efficient contention-based MAC protocol for the optical-acoustic underwater wireless sensor network, called OA-CMAC. OA-CMAC protocol transmits data after successful acoustic and optical handshakes. It can effectively improve the network throughput and channel utilization. Nevertheless, the handshake mechanism aiming at reserving channel and reducing collisions is inescapably required before data transmission launches, and the global propagation information is no exception.

Both Reinforcement Learning (RL) [40] and Deep Reinforcement Learning (DRL) [41] are intelligent algorithms which are capable of interacting with environments, learning the fast time-varying changes of environments, and choosing the appropriate actions. They have been used frequently to enhance the network performance with more effectiveness and lower complexity. Recently, the combination of underwater MAC protocols with intelligent algorithms has also attracted great attention [18,19,20,23,24]. As a value-based RL technique, Q-learning [42] is combined with a conventional MAC protocol in [43], where higher channel utilization has been acquired through trial-and-error. Nevertheless, once the Q-Table is deposited with overloaded Q values, Q-learning may learn and converge tardily or even diverge. In order to address the inadequacies in training, Deep Q-network (DQN) algorithm has been proposed in [22] by combining Q-leaning with Deep Neural Network (DNN) [44]. To address the overload Q-Table, Q-learning supplies target values for Deep Q-Neural Network. DQN fits the Q-values in Q-Table, and it solves the instability as well as divergence of reinforcement learning in large state space and continuous action space. Thus, DQN can learn successful policies from high-dimensional sensory inputs. Two vital technologies, including Experience Replay and Fixed Target Network are involved to better train models in the DQN model [45].

DQN has been widely exploited in [23,24] for the design of underwater MAC protocols to improve the network performance. Most of the pre-existing MAC protocols are designed for a homogeneous system in which all the network nodes adopt identical MAC protocols. Few studies of the existing MAC protocols concentrate on heterogeneous networks, where sensor nodes employ diverse MAC protocols. In reality, heterogeneous systems can reflect the real underwater environment more closely, since the underwater environment may consist of various Unmanned Aerial Vehicles (UAVs), submarines, Remotely Operated Vehicles (ROVs), and so forth [46], where multiple source nodes may utilize different MAC protocols. The introduction of heterogeneous architecture is of great and positive realistic significance in UWSNs. Furthermore, it has the potential to promote the development and application of Internet of Underwater Things (IoUT) [47].

The authors in [48] do not merely integrate the deep reinforcement learning algorithm with conventional MAC protocols of Wireless Sensor Networks (WSNs); meanwhile, they also introduce the heterogeneous network framework when constructing the Deep Reinforcement Learning Multiple Access (DLMA) network. Source nodes applying different slotted MAC protocols attempt to transmit packets to the relay node, and the node using the DLMA protocol is referred to as an agent node. Thanks to the existence of multiple time-slotted protocols, time slot allocation can be readily implemented. Through a series of observation–action rewards, the agent nodes can learn to take the proper actions to acquire the idle time slots. Therefore, the agents can achieve near-optimal performance with respect to the objective, even without knowing the detailed operating mechanisms of other coexisting MAC protocols. It is demonstrated that sum throughput can be easily maximized by combining deep reinforcement learning with MAC protocols in heterogeneous networks.

Even though DLMA is only applicable to heterogeneous Terrestrial Wireless Sensor Networks (TWSNs), its exceptional performance in terrestrial areas has garnered considerable attention in MAC design for acoustic UWSNs. Both [24] and [23] exploit the same heterogeneous system model of acoustic UWSNs to design feasible DRL MAC protocols. The sensor nodes using the two DRL-based protocols can fully capture the available time slots caused by long latency or the free time slots unused by other nodes. Optimal network throughput can thus be achieved without knowing the propagation delays and transmission strategies of non-agent nodes. However, the DR-DLMA protocol and the DRL-based MAC in [24] merely consider the single acoustic channel for transmissions.

With the DRL algorithm, Reference [49] puts forward a multi-channel deep-reinforcement learning multiple access protocol (MC-DLMA) for heterogeneous TWSNs, expediting more efficient spectrum utilization. By learning the transmission modes of the existing radio nodes, the MC-DLMA node can fully utilize underutilized spectrum resources and maximize network throughput. However, this protocol cannot be directly adopted in UWSNs without modification due to the harsh underwater environment. 

On balance, the introduction of the heterogeneous structure, where sources nodes use different MAC protocols to communicate with a relay node, will initially assist UWSNs with near-realistic underwater acoustic network models. In addition, the deep reinforcement learning-based MAC protocols will guarantee performance enhancement for the heterogeneous system. Moreover, the alliance of optical and acoustic transmissions has been demonstrated to effectively improve the network performance. Unfortunately, to our best knowledge, this promising hybrid technology has not been applied to the heterogeneous UWSNs to design corresponding MAC protocols.

In light of the analysis above, we propose a DRL-based hybrid optical and acoustic dual-channel MAC protocol for a heterogeneous underwater framework. First, we provide an efficient heterogeneous network model by integrating optical and acoustic transmissions, which permits source nodes to send packets to the relay node with different slotted MAC protocols. The alliance of optical and acoustic technologies can further enable nodes to deliver packets in either optical or acoustic mode. The advantages of rapid transmission and high bandwidth of the optical mode and the stable and long-range transmission of the acoustic mode will be combined effectively. In addition, by joining with deep reinforcement learning, the agent nodes can assist in capturing and compensating underutilized channels that are not sufficiently occupied by the non-agent nodes. Furthermore, a distinct reward policy has been adopted to distinguish between the transmissions of optical and acoustic channels. Consequently, priority compensation can be applied to the optical channel with a higher data transmission capability. Eventually, the objective of maximizing the throughput and channel utilization will be achieved by the continuous learning and training of the agents. 

In Table 1, we summarize and compare our model with similar models proposed by other authors.

## 3. Model of DQN

Since deep Q network derives from fundamental Q-learning, we first introduce the Q-learning paradigm in this subsection. Next, the deep reinforcement learning that includes the deep Q network will be explained. Moreover, the basic contents and learning processes of deep reinforcement learning will be explained, which can lay the foundation for the combination of deep reinforcement learning and data transmissions of the MAC protocol in Section 4.

### 3.1. Fundamental Q-Learning Model

**Figure 1:**
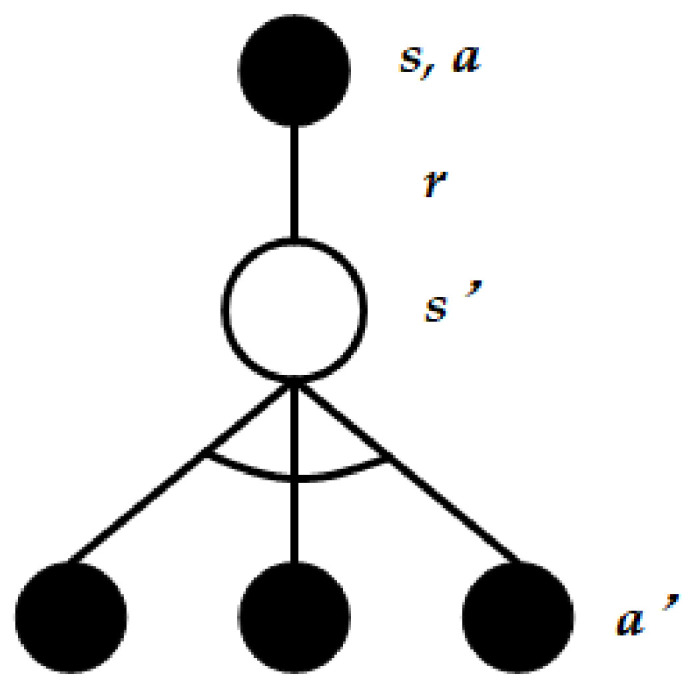
Simple model of Q learning.

Q-learning [40] is a prevailing reinforcement learning algorithm with the main components of <*a_t_,s_t_,r_t_*>, where *a_t_*, *s_t_*, *r_t_* are the set of the action taken at *t*, the environmental state at *t*, and corresponding reward, respectively. The environmental state becomes *s_t+1_* after receiving the reward *r_t_*. Unlike the model-based algorithm with a priori knowledge of the environment model, the transition probability matrix is not required in the model-free Q-learning [50]. Moreover, this approach is an off-learning type which applies two control strategies for choosing new actions and updating Q value function, respectively. The action choosing policy is exactly the greedy algorithm explained in further detail below. As illustrated in Figure 1, the agent is in the state *s* and executes the action *a* with a certain probability according to the *ε*-greedy method. After that, the agent will get the reward *r* and upgrade the environment state *s* into *s′*. *ε*-greedy algorithm is exploited to choose the optimal action *a′* and update the Q value function as follows:

(1) $$Q\left(s_{t},a_{t}\right)\leftarrow Q\left(s_{t},a_{t}\right)+\alpha \left[r_{t+1}+\gamma \underset{a^{\prime }}{\max}Q\left(s_{t+1},a^{\prime }\right)-Q\left(s_{t},a_{t}\right)\right]$$

In Equation (1), α is the learning rate of the agent, and γ is the discount factor to reward. The *ε*-greedy algorithm can be described as:

(2) $$a=\left\{\begin{array}{l} \arg\max_{a^{\prime }}Q\left(s,a^{\prime }\right),\;\mathrm{with}\;\mathrm{probability}\;1-\varepsilon \\ a\;\mathrm{random}\;\mathrm{decision},\;\mathrm{with}\;\mathrm{probability}\;\varepsilon \end{array}\right.$$

In Equation (2), the behavior is selected by an *ε*-greedy policy that follows the greedy policy with probability 1-*ε* and selects a random action with probability *ε*. The former branch is usually applied to choosing actions on the premise of acquiring maximal Q value according to the current state and getting the new action and reward after the current execution. This step is called target policy. However, the maximal Q value does not always result in the optimal action. *ε*-greedy can help to reduce the chances of taking the wrong actions. The latter branch is applied to choosing actions randomly for exploration, which is also called behavioral policy. As we can see, the *ε*-greedy is targeted at solving the balance between exploration and exploitation, where *ε* is set to a larger value initially for abundant exploration and it gradually decreases for sufficient exploitation.

Q Table is created to store $Q\left(s,a\right)$ of each state *s* and action *a*, where values of rows represent state space and values of columns represent action space. Initially, a primal Q Table is set according to Equation (3), where all the Q values are 0, because the whole system is at initialization. We set this initial Q-Table to briefly describe the basic components of Q-Table.

(3) Qs,a=0000as

At each time-step, the first state is initialized randomly. Next, a corresponding action *a* will be executed on the basis of Q Table in current state *s* with *ε*-greedy. After performing action *a*, the model can get a state *s′* and current reward value *r*, following by the updating of $Q\left(s,a\right)$ and sequential loops. The whole algorithm of Q-learning determines an optimal policy to obtain a higher cumulative discount reward in a step-by-step iteration manner [51].

When the action space is finite, Q learning usually performs efficiently in finding a policy by learning the optimal value function implicitly because action optimization can be done by exhaustive enumeration method [52]. By contrast, in problems with continuous and large-scale action spaces, performance of Q learning may be greatly reduced and even diverge. Therefore, Deep Q Learning (DQL) is proposed in [22] to deal with emerged problems of Q learning.

### 3.2. Deep Q Learning

Deep Q learning exploits Q learning with continuation and extension, in which a nonlinear function approximator (i.e., the deep neural network) is used to approximate Q value function instead of Q Table [22]. The function approximator with weights *θ* is described as Q-network: $Q\left(s,a\right)=Q\left(s,a;\theta \right)$. To obtain an unbiased estimator of the mean-squared Bellman error while training the Q-network [53], the target Q-network is utilized to update the values of the primary Q-network periodically and slowly in order to figure out the instabilities in training process [54]. It is almost identical to the original Q-network structure, except for the parameter *θ*. The target network does not interact with the environment, and it does not renew in each time-step immediately either. The target network is actually synchronized with the Q-network after a certain number of iterations (*F* time slots) by replacing $\theta^{-}$ with *θ*. The Q-network and loss function defined in Equation (4) updates continuously in DQN update training, until the loss function converges to a minimum value. Loss function is used to minimize the mean-squared Bellman error with respect to the old parameter $\theta^{-}$ at each update irritation. The current parameters *θ* are updated by a Stochastic Gradient Descent (SGD) algorithm [55].

(4) $$L\left(\theta \right)=E\left[{\left(r+\gamma \underset{a^{\prime }}{\max Q\left(s^{\prime },a^{\prime };\theta^{-}\right)-Q\left(s,a;\theta \right)}\right)}^{2}\right]$$

Another key technique for achieving stability in update processes is experience replay, because samples in deep learning are required to be mutually independent and uniformly distributed to each other, while the observations of original *Q* learning are correlated with each other and do not satisfy this condition. Experience replay [56] helps the training process to achieve stability and break the temporal dependency among the observations, which are employed to train the deep neural network. The key idea of experience replay is to train the agent with the transitions sampled from the buffer of previously experienced transitions [57]. At each time-step *t*, after the agent interacts with the environment, it stores the corresponding experience tuple *e_t_* = (*a_t_*,*s_t_*,*r_t_*,*s*_*t*+1_) into the replay memory, where *a_t_*, *s_t_*, *r_t_*, *s*_*t*+1_ are the set of the state input at time *t*, the action selected at time *t*, corresponding received reward, and the next state transited from *s_t_* after taking *a_t_* at time (*t* + 1), respectively. The replay memory D consists of multiple experience tuples, i.e., D = {*e*_1_,*e*_2_,…,*e_M_*}, where *M* is the state history size [58]. At each iteration of DQN, a mini-batch of states, actions, rewards, and next states are sampled from the replay memory as observations to train the Q-network, which approximates the action–value function [53], and DQN adopts the *ε*-greedy strategy to generate experiences. In addition, the original experience tuple will be deleted as long as the replay memory becomes saturated, and the latest experience tuple will store in the replay memory.

## 4. System Model

**Figure 2:**
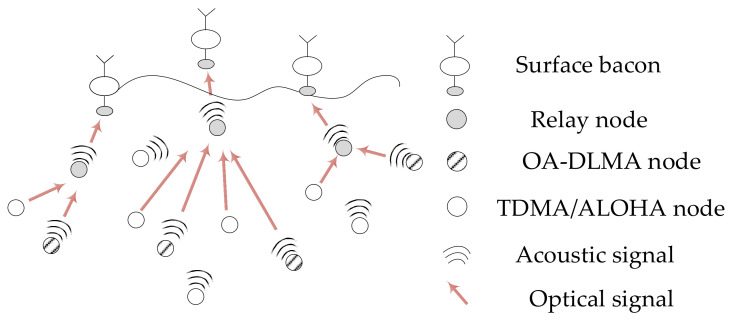
A heterogeneous UWSN consists of multiple source nodes and relay nodes, where source nodes can transmit data packets to the relay node through optical or acoustic channels.

The heterogeneous UWSN architecture with hybrid optical and acoustic dual channels considered in this paper is illustrated in Figure 2, which is mainly formed from underwater sensor nodes (i.e., multiple source nodes and one relay node), underwater uplink acoustic and optical channels, and several water surface bacons. The relay node is equipped with an acoustic–optical transceiver. Source nodes are randomly scattered around the relay node’s maximum one-hop communication coverage and send data packets towards the relay node via optical or acoustic channels. There is at least one sensor node adopting our proposed OA-DLMA protocol, and a node with the OA-DLMA protocol is regarded as an agent node. Each agent node is also equipped with an acoustic–optical transceiver, and can transmit data packets by adaptively choosing an optical channel or an acoustic channel to maximally utilize the underutilized channels, while other non-agent source nodes employing TDMA or ALOHA protocols are equipped with an acoustic transceiver or an optical transceiver in order to save energy. TDMA nodes only deliver data packets at the beginning of each time slot in a distributed manner. ALOHA nodes are actually q-ALOHA nodes, which transfer data packets with certain transmission probabilities at the beginning of each time slot. In other words, the non-agent nodes are restricted to sending packets on a specific channel. An agent node, unlike non-agent nodes, is not allotted a dedicated channel for its packet transmissions, and it can only make use of the underutilized channels of non-agent nodes. For a specific time slot, the agent can only compensate for the unused slot on an optical channel or an acoustic channel. It cannot transmit on both optical and acoustic channels in the same slot. Because of the high-speed transmissions, each agent is encouraged to prefer compensating the unused time slot on the optical channel. Collisions will occur if agent and non-agent nodes transmit across the same channel at the same time. The relay node returns an acknowledgement packet as a response to the data packet from the source node, and the same channel as the transmitting channel of the source node is adopted for replying. It forwards the collected information to the surface bacons via optical or acoustic channels for subsequent communication in the terrestrial area. The relay node can also occupy the optical and acoustic channels concurrently, if necessary, because the two types of communication do not bother each other.

In our heterogeneous underwater system, we divide the time into fixed-length frames. Each frame is further split into smaller and equal time slots. Similar to [23], data packet delivery time is generally fixed at the beginning of each time slot. The short acknowledgement packets are sent by the relay node afterwards. Additionally, the same type of packet for different nodes has the same packet length. One slot length consists of two segments for data transmission and acknowledgement, respectively. The relay node can only receive one data packet in a single data transmission. Once more than one data packet arrives at the relay node simultaneously, conflicts ensue. Besides, conflicts may still happen if more than one node attempts to take up the same channel. After the relay node receives the data packet at the current time slot, it will send back an ACK acknowledgement to the sender at the current time slot. The ACK packet will be received by the sender at this time slot because the slot length is long enough to accommodate a complete transmission.

In the hybrid optical–acoustic dual channel transmission mode, because of the non-negligible propagation delay caused by slow acoustic velocity, the acoustic mode slot length should be sufficiently long to cover the entire transmission between the source node and the relay node. The slot length should be no less than two times the maximum propagation delay (*T_max_delay_*) plus one duration for transmitting acoustic data information (*T*_1_) and one duration for transmitting an acoustic ACK acknowledgement (*T_C_*), that is:

(5) $$t_{slot}\geq 2\cdot T_{max\_delay}+T_{1}+T_{C}$$

Besides, the duration of optical and acoustic ACK acknowledgements can be regarded as the same, since the data size of ACK packets is both small and their values have little difference. The optical mode maintains a time slot length as in Equation (6) that holds a duration for transmitting optical data information (*T*_2_) plus a duration for an optical ACK signal (*T_C_*), since the optical propagation delay can be almost neglected.

(6) $$t_{slot}\geq T_{2}+T_{C}$$

Because both acoustic and optical transmissions exist in one common network model, two types of time slot length are assumed to be the same, which satisfies Equation (5). According to Equations (5) and (6), it is obvious that in each time slot, optical data transmission time is much longer than that of acoustic data transmission. In addition, due to the high optical bit rate, source nodes can transmit more data bits by optical channel during one time slot. In the real traffic case, high-speed transmission of data (such as pictures, videos, etc.) [59] is guaranteed by the optical transmission technique.

**Figure 3:**
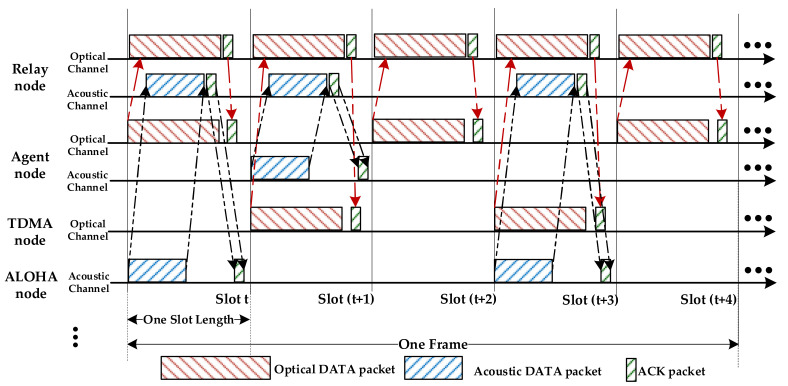
Slotted operations of two non-agent nodes and one agent node towards one relay node: One of the non-gent nodes uses an optical channel to send data packets, another occupies an acoustic channel for packet transmissions. They do not interfere with each other. The agent can utilize the unused time slots for transmissions.

The non-agent nodes using the TDMA protocol transmit packets in specific time slots within a frame through a fixed allocation method. The nodes employing the ALOHA protocol are assigned a transmission probability at the fixed sending time [60]. We assume that some non-agent nodes can only send data packets in an optical channel, and others can transfer data packets through an acoustic channel, thus they do not interfere with each other in this way. Similarly, equipped with an acoustical-optical transceiver, each agent node can launch communication with the relay node via an optical channel or an acoustic channel at the same time. Combining with the deep reinforcement learning algorithm, the agents can interact with the environment and take full advantage of the underutilized channels (optical and acoustic channels) after a series of observations and actions. Namely, the agents will capture the available time slots of hybrid optical and acoustic channels no matter which channel is underutilized. Therefore, the network performance can be improved. A fundamental example of slotted operations for a heterogeneous UWSN with two non-agent nodes and one agent node is illustrated in Figure 3.

In Figure 3, the non-agent nodes (one TDMA node and one ALOHA node) transmit their own data packets at the beginning of the time slots to the relay node by optical channel or acoustic channel, respectively. Specifically, the TDMA node adopts time slot (*t* + 1) and time slot (*t* + 3) to initiate sessions with the relay node. The ALOHA node adopts slot *t* and slot (*t* + 3) for transmitting data packets to the same relay node. Once the relay node acquires the data packet, the ACK packet backtracks to the corresponding sender immediately, and the acknowledgement can be received within the same time slot. In order to maximize the throughput and channel utilization of the overall network, the agent is not deliberately assigned an uplink channel. That is, it will learn the transmission manner of the non-agent nodes and utilize the vacant time slots of underutilized channels. As depicted in Figure 3, the agent acquires the vacant time slots of the optical and acoustic channels. It occupies the slot *t*, slot (*t* + 2), and slot (*t* + 4) of optical channel that are not used by the TDMA node to send data packets. Meanwhile, the agent also occupies the unused slot (*t* + 1) of the acoustic channel to deliver data packets to the relay node. By capturing more available time slots of an optical or acoustic channel, the agent can transmit more data packets to the relay node. Correspondingly, the network performance improvements in terms of throughput and channel utilization can be achieved with our proposal.

## 5. OA-DLMA Protocol

In this section, we discuss the data transmission manner of the hybrid optical and acoustic dual channel MAC protocol in our slotted heterogeneous system as a DRL problem. The agent attempts to occupy the time slots that are not occupied by non-agent nodes of different channels. Specifically, we assume that the current state is in the time slot *t*. Related definitions of deep reinforcement learning are explained as follows.

**Agent:** A node using the OA-DLMA protocol is referred to as an agent. Agents are capable of interacting with underwater heterogeneous environments and choosing appropriate actions.

**Action:** We suppose that there are two available channels, including an optical channel and an acoustic channel. *a_t_* defines the selected behavior by the agent at time step *t*: $a_{t}\in \left\{0,1\right\}$ is the action space. *a_t_* = 0 means that the agent chooses not to send in the current time slot *t*. *a_t_* = 1 means that the agent chooses to send in time slot *t* on the channel *i*, $i\in \left\{1,2\right\}$, in which *i* = 1 represents the agent sends packets via acoustic channel, and *i* = 2 denotes the agent chooses the optical channel for data transmission.

**Observation:** After the agent behaves an action at time slot *t*, it will acquire related observation information of the dual channels. If it decides to send a data packet towards the relay node via channel *i* at time slot *t*, it will receive the acknowledgement from the relay node at time slot *t* along with the ACK packet. The observations of the agent are presented in Equation (7):

(7) $$o_{t}=\left[o_{t}^{1},\;o_{t}^{2}\right]$$

where each element represents the individual observation of a particular channel. If *a_t_* = 0, as for any $i\in \left\{1,2\right\}$, the observation $o_{t}^{i}=\mathrm{Success}\;\mathrm{or}\;\mathrm{Idleness}$ expresses whether the transmission of non-agent nodes on channel *i* is successful or the channel *i* is not used at time slot *t*. If $a_{t}=i\in \left\{1,2\right\}$, $o_{t}^{i}=\mathrm{Success}\;\mathrm{or}\;\mathrm{Failure}$ indicates whether the transmission of the agent succeeds or not on channel *i*.

**State:** After selecting action *a_t_*, the system state varies from the past state to the next state. *s_t_* represents the environment state at time slot *t* and are constituted by observations and actions. Specifically, in time slot *t*, the action–observation pair is as follows on receiving the observation value *o_t_*.

(8) $$z_{t}≜\left(a_{t},o_{t}\right)$$

Thus, the state in slot *t* can be defined as:

(9) $$s_{t}≜\left(z_{t+1-M},\ldots ,z_{t}\right),$$

where *M* is the state history length, implying that the current system involves *M* action–observation pairs from slot (*t* + 1 − *M*) to slot *t*.

**Reward:** After selecting action *a_t_* to transfer from *s_t_* to *s_t+1_*, the agent can obtain the corresponding reward *r_t_* of the dual channel in our underwater system. To further improve the network performance, priority compensation can be considered for the optical channel since it can undertake more data messages. We adopted a distinguishing reward policy, and the rewards of each channel are defined as:

(10) $$r_{t}^{i}=\left\{\begin{array}{ll} 0, & o_{t}^{i}=\mathrm{Idleness}\;\mathrm{or}\;\mathrm{Failure}\\ 1, & o_{t}^{i}=\mathrm{Success}\;\mathrm{and}\;i\;\mathrm{is}\;\mathrm{acoustic}\;\mathrm{channel}\\ 2, & o_{t}^{i}=\mathrm{Success}\;\mathrm{and}\;i\;\mathrm{is}\;\mathrm{optical}\;\mathrm{channel} \end{array}\right.$$

In Equation (10), $r_{t}^{i}=0$ means that agent does not take action or collisions happen if the agent sends packets; $r_{t}^{i}=1$ represents that the agent sends packets on the acoustic channel successfully; $r_{t}^{i}=2$ shows that the agent delivers packets on the optical channel successfully. We set different values of reward to distinguish the behavior on the optical channel and the acoustic channel. In this way, the agent can be qualified to prioritize the compensation of optical transmission. By aggregating the rewards of hybrid optical and acoustic channels, the actual accumulated reward of the agent is given by $r_{t}=\sum_{i=1}^{2}r_{t}^{i}$, which merges the rewards of optical channel and acoustic channel.

**Experience Replay:** Experience replay is introduced to break the correlations between samples and therefore reduces the variance of the updates. At each time-step *t*, after the agent has interacted with the underwater environment, it stores the corresponding experience tuple *e_t_* = (*a_t_*,*s_t_*,*r_t_*,*s*_*t*+1_) into replay memory D = {*e*_1_,*e*_2_,…,*e_M_*}. After storing enough tuples, the system will launch experience replay to select random tuples for continually renewing rewards and states. The loss function in the subsequent content is exactly calculated according to the values extracted from experience replay.

**Fixed Target Network:** The target Q-network $Q\left(s,a;\theta^{-}\right)$ with parameter $\theta^{-}$ is used to update the primary Q-network’s values frequently, but slowly, in order to figure out the instabilities in training process.

**Figure 4:**
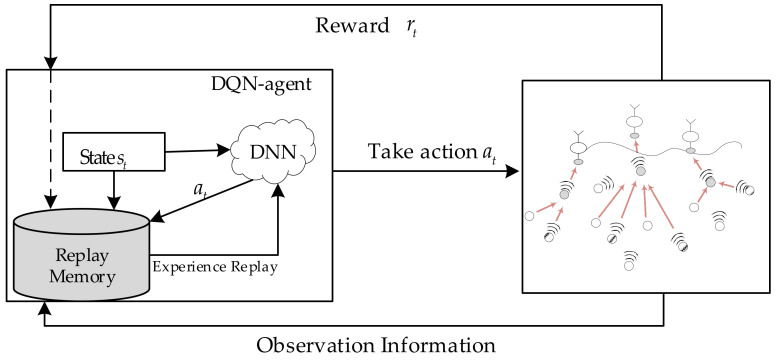
Deep Q network for the OA-DLMA protocol of UWSNs.

The whole operation of the OA-DLMA protocol is shown in Figure 4. We employ DQN algorithm to address the hybrid optical–acoustic MAC design problem in this paper. Based on policy *π*, the agent node executes action *a_t_* when the environmental state is *s_t_*. The corresponding Q value is described as $Q^{\pi }\left(s_{t},a_{t}\right)$. The updating of Q value is an iterative process of weighted averaging of past Q values and future information. In the OA-DLMA system, the update of Q values follows Equation (11):

(11) $$Q\left(s_{t},a_{t}\right)\leftarrow Q\left(s_{t},a_{t}\right)+\alpha \left[r_{t}+\gamma \;\underset{a^{\prime }}{\max}Q\left(s_{t+1},a^{\prime }\right)-Q\left(s_{t},a_{t}\right)\right]$$

The loss function here is modified as:

(12) $$L\left(\theta \right)={\left[r_{t}+\gamma \;\underset{a^{\prime }}{\max}Q\left(s_{t+1},a^{\prime };\theta^{-}\right)-Q\left(s_{t},a_{t};\theta \right)\right]}^{2}$$

In Equation (12), the third term is the eval Q value, and the first two terms on the right are the output of target-network, i.e.,

(13) $$y_{r_{t},s_{t+1}}=r_{t}+\gamma \;\underset{a^{\prime }}{\max}Q\left(s_{t+1},a^{\prime };\theta^{-}\right)$$

Equation (13) is updated through Stochastic Gradient Descent (SGD) to get the *θ* value as follows.

(14) $$\theta \leftarrow \theta -\rho \left[y_{r_{t},s_{t+1}}-Q\left(s_{t},a_{t};\theta \right)\right]\nabla Q\left(s_{t},a_{t};\theta \right)$$

in which ρ is the time slot number requested at each iteration.

The learning and training process of OA-DLMA is described in Algorithm 1.
**Algorithm 1:** Training process of the OA-DLMA protocol for heterogeneous UWSNs.1: Initialize *α*, *γ*, *D*, *ε*, *F*, *M*, *N_E_*   //*F* is the update frequency of the target network2: Initialize Q-network and target-network with random weights *θ*, $\theta^{-}$3: Initialize state randomly4: **for** each time slot *t*
**do**5:  Input current state *s_t_* into Q-network and output Q value $Q\left(s_{t},a_{t};\theta \right)$;6:  Select an action *a_t_* using Equation (2);7:  Get *o_t_* through collecting $o_{t}=\left[o_{t}^{1},o_{t}^{2}\right]$.8:  **for**
*I* = 1 to 2 **do**9:   **if**
$0\leq t\leq t_{slot}$
**then**10:    $s_{t+1}=s_{0}$
11:   **else**12:   **if**
$o_{t}^{i}=\mathrm{Idleness}\;\mathrm{or}\;\mathrm{Failure}$
**then**13:    $r_{t}^{i}=0$.14:   **else if**
$o_{t}^{i}=\mathrm{Success}\;\mathrm{and}\;i\;\mathrm{is}\;\mathrm{optical}\;\mathrm{channel}$15:    $r_{t}^{i}=2$.16:   **else**17:    $r_{t}^{i}=1$.18:   **end if**19:   **end if**20:   Get the reward $r_{t}$ through collecting $\left\{r_{t}^{1},r_{t}^{2}\right\}$;21:   Generate the next state *s*_*t*+1_ based on Equation (9);22:   Store experience *e_t_* = (*a_t_*,*s_t_*,*r_t_*,*s*_*t*+1_) into replay memory D;23:  **end for**24:  Calculate the short-term average rewards as Equation (15);25:  Calculate the channel utilization as Equation (16);26:  Select random sample minibatch of experience tuples from D;27:  Train Q-network;28:  Compute loss function by Equation (12);29:  Perform SGD to minimize loss function;30:  Update *θ*;31:  Every F time slots copy current Q-network to target-network: $\theta^{-}=\theta$;32: **end for**

As given in Algorithm 1, it reveals that this algorithm will traverse all the *n* time slots to reach convergence. In the process, learning and training will also be executed over two channels to obtain corresponding observations and rewards for optimizing network targets. As a result, the complexity of this algorithm is $O\left(n\right)$, since it only needs to walk through all the time slots. Namely, the algorithm complexity running time nearly depends on the ergodic total time slots and exhibits linear growth.

## 6. Performance Evaluation

### 6.1. Simulation Setup

In this section, we evaluate the performance of the proposed OA-DLMA protocol by comparing it with two representative and promising protocols, OA-CMAC [39] and MC-DLMA [49], in heterogeneous hybrid optical and acoustic underwater sensor networks. OA-CMAC is a handshake-based MAC protocol that combines optical and acoustic transmission techniques to save energy. It can transmit large data messages (videos, pictures, etc.) over a short distance and send small-sized data packets over a long range. MC-DLMA is a multi-channel protocol for heterogeneous terrestrial wireless sensor networks based on DRL. It divides the wireless spectrum resource into several sub-channels, and each non-agent node sends packets to the relay node on its pre-allocated channel. Since the MC-DLMA performs well in terrestrial systems, we tailor it to our underwater system with the assumption that it can send data packets through optical and acoustic channels, denoted as UMC-DLMA. The inherent training mechanism of MC-DLMA is followed by UMC-DLMA. It does not deliberately distinguish between specific actions on the optical and acoustic channels, and the same reward policy is set for both. In the following simulations, we first consider the scenario where one agent (OA-DLMA node) coexists with one TDMA node and one ALOHA node. The three nodes transmit data packets to the relay node. All the nodes adopting different MAC protocols are required to send their packets only at the beginning of each time slot. Besides, we further consider an extended scenario including more agents and non-agent nodes with optical and acoustic channels. 

All the simulations are based on Python (Version 3.5.4) and Ubuntu 16.04 LTS platforms, and Tensorflow (Version 1.6.0) [61] and Keras (Version 2.4.3) [62] are adopted as the software frameworks for deep reinforcement learning. The activation function is RELU [63]. The discount factor γ is set to 0.9, and the state history length *M* is 32. The greedy exponent *ε* is initially set to 1 in *ε*-greedy algorithm, then it decays by 0.995 every time slot until it reaches a minimum value of 0.01. The capacity of replay memory D is 560, and the number of extracted sample tuple *N_E_* is 32 at each training session. The update frequency *F* is 480 for overwriting the old parameter $\theta^{-}$. All the hyper-parameters and parameters are summarized in Table 2 and Table 3, respectively.

### 6.2. Simulation Metrics 

In this paper, we assess the performance of our OA-DLMA protocol in terms of short-term sum throughput [48] and channel utilization [18].

The short-term sum throughput is defined as the average packet rate that is successfully received by the relay node during the measurement duration (i.e., the duration of the past *N_w_* time slots after the network converges), which is defined as:

(15) $$\mathrm{Throughput}=\frac{T_{1}\cdot acoustic\;data\;rate\cdot N_{1}+T_{2}\cdot optical\;data\;rate\cdot N_{2}}{\mathrm{Measurement}\;\mathrm{duration}}\;bit/s,$$

where *T_i_* (*i* = 1,2) represents the duration to send an acoustic data stream or optical data stream in one time slot, respectively. *N_i_* (*i* = 1,2) denotes the number of time slots that are used by acoustic transmissions or optical transmissions during the whole measuring duration. For ease of description, sum throughput and throughput are used interchangeably with short-term sum throughput in the following.

The channel utilization is defined as the proportion of the time for all the channels to transmit acoustic and optical data packets over the total measurement duration, which can be calculated as:

(16) $$\mathrm{Channel}\;\mathrm{Utilization}=\frac{T_{1}\cdot N_{1}+T_{2}\cdot N_{2}}{\mathrm{Mesaurement}\;\mathrm{duration}\cdot C},$$

where *C* is the number of used channels.

### 6.3. Simulation Results

#### 6.3.1. The Coexistence of One OA-DLMA Node with One TDMA and One ALOHA Node

We first evaluate the MAC performance under the coexistence of one agent node (OA-DLMA node) with two non-agent nodes (one TDMA and one ALOHA node) in two channels (one optical channel and one acoustic channel). The two non-agent nodes can alternate between optical and acoustical transmission technologies. We consider two simulation scenarios: (a) The TDMA node delivers packets to the relay node by acoustic channel, and the ALOHA node sends packets to the relay node through an optical channel; (b) The TDMA node sends packets to the relay node by optical channel, and the ALOHA node transfers packets to the relay node through an acoustic channel. In more detail, the number of time slots that are occupied by the TDMA node varies from 1 to 10, and the transmitting probability of the ALOHA node increases from 0.1 to 1. We let *R* and *q* denote the number of time slots occupied by the TDMA node within 10 slots and the transmission probability of the ALOHA node, respectively. In the two scenarios, we first let *R* be fixed at 5 and *q* be variable from 0.1 to 1 with a step size of 0.1. Afterwards, *q* is fixed at 0.6 and *R* varies from 1 to 10.

To verify the algorithm convergence and theoretical validity, we also exhibit the optimal values of every scenario. Of particular note is that the theoretical optimal values in the following content are all deduced under the circumstance where the available time resources are maximally utilized by source nodes. The related derivations are all explained in Appendix A.

**Figure 5:**
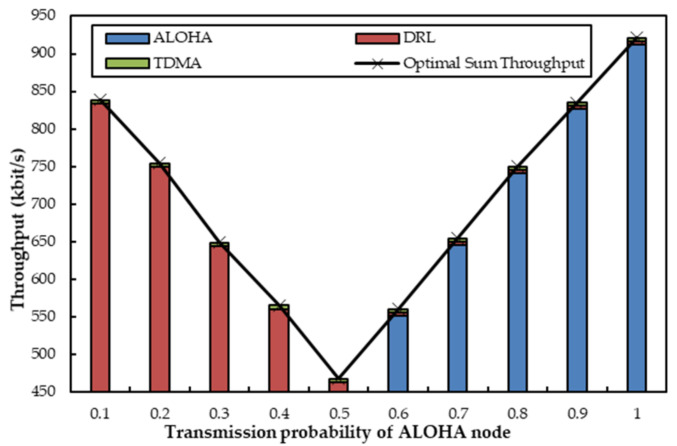
Sum throughput when one agent coexists with one optical ALOHA node and one acoustic TDMA node, which have fixed *R* and variable *q*, respectively.

(a) *Acoustic TDMA and Optical ALOHA*

(1.) *Short-term Sum Throughput:* Figure 5 depicts the sum throughput when one agent coexists with one ALOHA node and one TDMA node. The TDMA node and the ALOHA node transmit data packets over an acoustic channel and an optical channel, respectively. The transmission probability *q* of the ALOHA node varies from 0.1 to 1, and the TDMA node occupies five time slots for data sending. From Figure 5, we can observe that the throughput of the TDMA node remains constant since it transmits packets through an acoustic channel with a fixed number of time slots. We can also see that the ALOHA node has little transmission since its transmission probability is relatively low when *q* is less than 0.5. The agent, thereupon, acts as a compensator to send packets with its transmission probability (1-*q*) in the current time slots as ALOHA should have behaved. When *q* is greater than 0.5, the ALOHA node launches optical transmissions more frequently, and the agent no longer transmits data on the optical channel, but on the TDMA node’s unused time slots in the acoustic channel. As a result, the throughput of the ALOHA node gradually becomes greater with the increase of *q*. Concurrently, the simulated throughput of the ALOHA node accords with the theoretical analyses in Appendix A.1. Furthermore, the ALOHA node can acquire optimal throughput when *q* is 1, and the optical channel is fully utilized by the ALOHA node at that moment. Another observation from Figure 5 is that the sum throughput can always stabilize at corresponding near-optimal values regardless of variations in *q*. The main reason is that the agent can help to utilize the underutilized slots of optical or acoustic channels.

**Figure 6:**
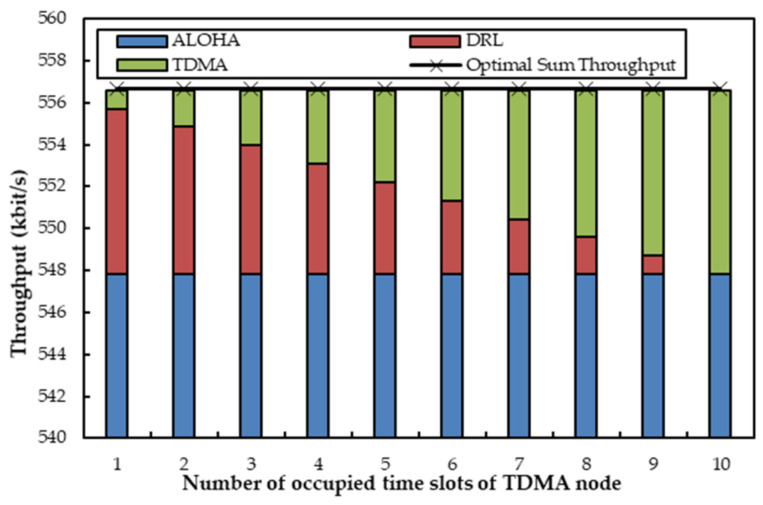
Sum throughput when one agent coexists with one optical ALOHA node and one acoustic TDMA node, which have fixed *q* and variable *R*.

**Figure 7:**
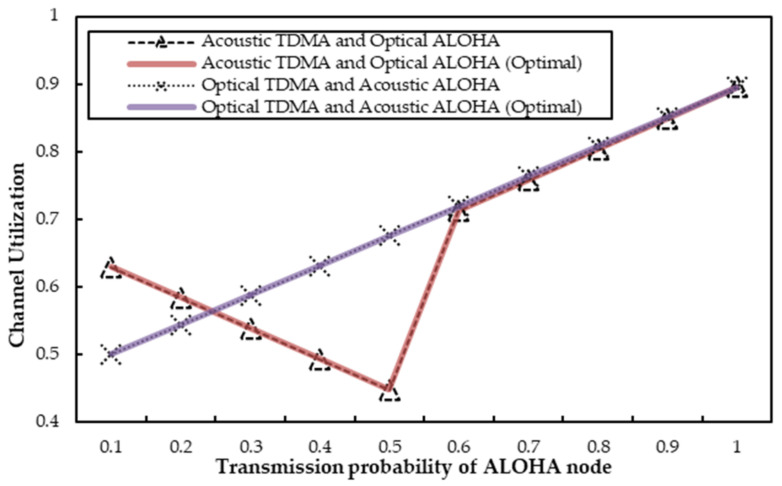
Channel utilization when one agent coexists with one ALOHA node and one TDMA node, which have fixed *R* and variable *q*.

**Figure 8:**
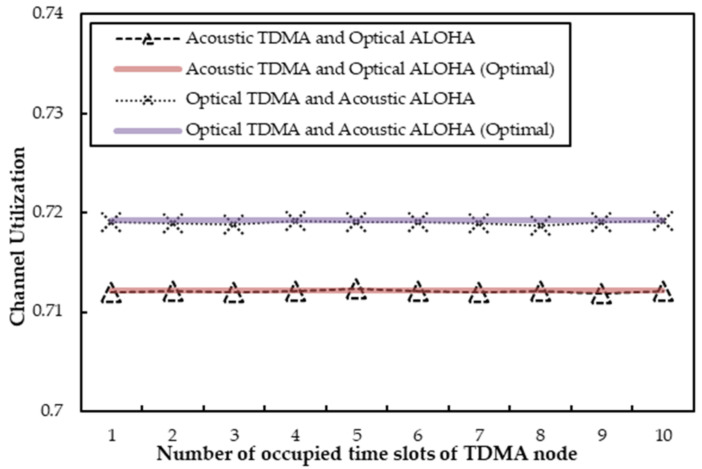
Channel utilization when one agent coexists with one ALOHA node and one TDMA node, which have fixed *q* and variable *R*.

Figure 6 depicts the sum throughput when one agent coexists with two non-agent nodes, where the transmitting probability of the ALOHA node is fixed at 0.6 and the TDMA node utilizes a changing number of time slots. In Figure 6, the throughput of the TDMA increases with the increase of available time slots till the upper limit number of time slots arrives. With the increasing use of time slots by the TDMA node, the throughput of the agent decreases. Since more time slots are occupied by the TDMA node, the agent has to utilize fewer time slots. The throughput of the ALOHA node nearly stays stable as the transmission probability *q* is fixed. It is also shown in Figure 6 that the sum throughput is always the same no matter how the TDMA node occupies time slots. The reason is that the agent can always apply the unoccupied time slots of the TDMA node in the acoustic channel. We can still observe that the total throughput can almost reach the optimal value. As a result, the agent is again proven to exhibit its capability to compensate for the insufficient transmissions within its abilities.

(2.) *Channel Utilization:* The corresponding channel utilizations are exhibited in Figure 7 and Figure 8, respectively. In Figure 7, the TDMA node utilizes a fixed number of time slots. The transmission probability of the ALOHA node varies from 0.1 to 1 simultaneously. As can be seen, the general channel utilization trend is an approximate “V” shape. In other words, with the increase of *q*, the channel utilization first decreases and then increases, and the best channel utilization can be gained when *q* = 1. The main reason is that the TDMA node always transmits on the acoustic channel with a fixed number of time slots. When *q* is no more than 0.5, the optical ALOHA node transmits less, and the agent replaces it to send on the optical channel with the transmission probability of (1 − *q*).

Thus, the channel utilization falls with an increase of *q* in this case. However, the agent does not send on the optical channel and instead captures and utilizes the underutilized slots of the TDMA node on the acoustic channel once *q* is greater than 0.5. In this case, the acoustic channel can be better utilized. As a result, the channel utilization enlarges with an increase of *q*. Accordingly, Figure 7 depicts the approximate “V” shaped trend of channel utilization. Besides, the simulation result accords with the analyses in Appendix A.1. In short, with the assistance of the agent, no matter what the value of *q* is, we can see that the channel utilization can still stay almost consistent with the theoretical optimal value.

Figure 8 shows the channel utilization when *q* is 0.6 and *R* varies from 1 to 10. We can see that the channel utilization remains quite stable in spite of the variation of occupied time slots by the TDMA node. Because the transmission probability of optical ALOHA is fixed at 0.6, the stable transmissions of the ALOHA node are far from influencing the channel utilization at this time. Except for this, the agent can acquire all the unused time slots of the TDMA node, and the slots can be sufficiently used. To sum up, the channel utilization will rarely change, since the transmissions by the acoustic and optical channels are quite stable. Meanwhile, it can also be observed from the figure that the simulated channel utilization basically stays consistent with the optimal value with the help of the agent.

**Figure 9:**
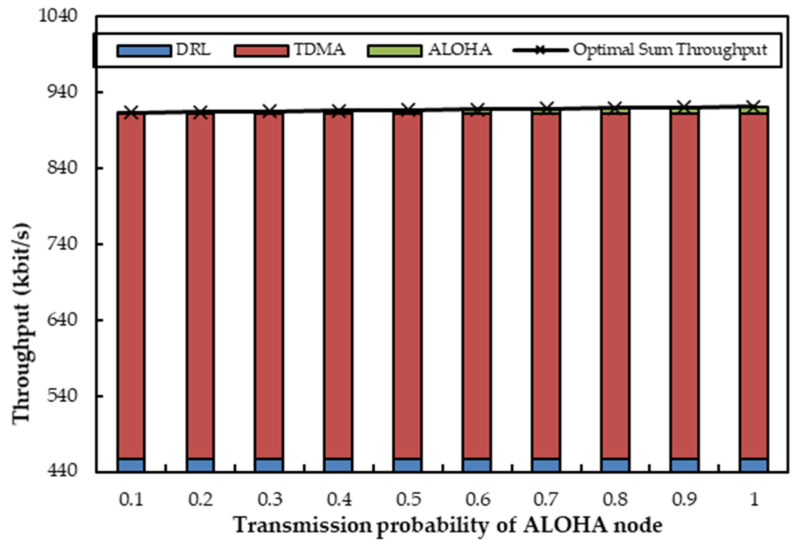
Sum throughput when one agent coexists with one acoustic ALOHA node and one optical TDMA node, which have fixed *R* and variable *q*.

**Figure 10:**
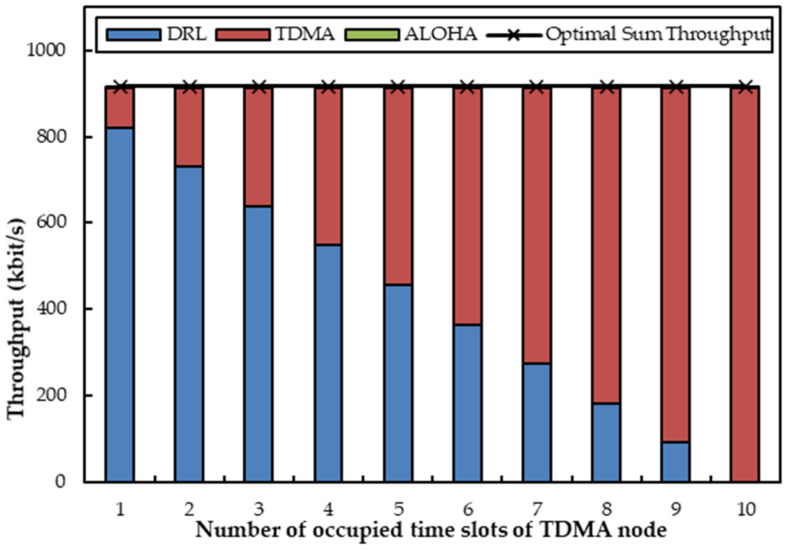
Sum throughput when one agent coexists with one acoustic ALOHA node and one optical TDMA node, which have fixed *q* and variable *R*.

(b) *Acoustic ALOHA and Optical TDMA*

(1.) *Short-term Sum Throughput:* Figure 9 and Figure 10 depict the sum throughput when one agent coexists with one acoustic ALOHA node and one optical TDMA node. First, *R* is fixed at 5, and *q* increases from 0.1 to 1 with a step size of 0.1. It can be clearly observed from Figure 9 that the throughput of the optical TDMA node is unchangeable, since it transmits packets through an optical channel with a fixed number of time slots. Because the agent can help to use the remaining slots of the TDMA node on the optical channel, its maximum throughput can be achieved. As for the ALOHA node on the acoustic channel, its throughput enhances with the increase of *q* because it transmits with the transmission probability *q*. The variation in the throughputs of the acoustic ALOHA node is not obvious because the acoustic node transmits less data than the optical node. The throughput of the agent remains constant since the agent can always compensate the unused time slots on the optical channel. As a result, the variation of the sum throughput is only influenced by the transmission of the ALOHA node. Undoubtably, the sum value still approaches the optimal value on account of the additional transmissions of the agent on the optical and acoustic channels.

With a fixed *q* and a change in *R* from 1 to 10, the throughput of the ALOHA node displays almost the same as in Figure 10. The throughput of the optical TDMA node scales up if it occupies more time slots, and the throughput of the agent scales down conversely. That is because the upper limit of the optical channel transmission ability is fixed. As a result, we can observe that the total throughput does not change when *R* changes, and it appears to be nearly the same as the optimal value. The phenomena are also regarded as the effects of the agent’s optimal actions. 

Summarized from Figure 5, Figure 6, Figure 9, and Figure 10, we have realized that the total throughput can always approach their optimal value even if nodes employ different technologies, not to speak of how *R* or *q* varies. That is because the agent can always take full advantage of the time slots of each channel via training and learning without any mutual interference.

(2.) *Channel Utilization:* Corresponding channel utilizations are described in Figure 7 and Figure 8. Obviously, the channel utilization displays the same trends as the total throughput value. In Figure 7, where the transmission probability *q* scales up from 0.1 to 1 and *R* is set to be 5, we can find that the channel utilization rises with the increasing *q*. The main reason is that optical transmissions are stable because the agent always helps the TDMA node with sufficient optical transmissions. Thus, the channel utilization is only related to the transmissions of the ALOHA node on the acoustic channel. The ALOHA node transmits more packets with a bigger value of *q*. Therefore, the channel utilization always increases until it reaches its maximum value. The simulated channel utilization is also very similar to the optimal channel utilization, due to the efficient compensation achieved by the agent node on the acoustic or optical channel.

In Figure 8, where the occupied time slots by the TDMA node scale up from 1 to 10, the channel utilization will not be affected anyway. That is due to the higher transmission probability of the ALOHA node (*q* = 0.6), as the agent has little chance to compensate for the ALOHA node on the acoustic channel at all. In general, the channel utilization in Figure 8 can nearly reach its optimal theoretical values as well, and the deviations between optimal values and simulated values can be nearly ignored. The reason is that the agent can acquire the unused time slots within its capacity and thus fully utilize the channel all the time, no matter how *R* or *q* changes. 

#### 6.3.2. The Coexistence of Multiple OA-DLMA NODES with Multiple TDMA and ALOHA Nodes

**Figure 11:**
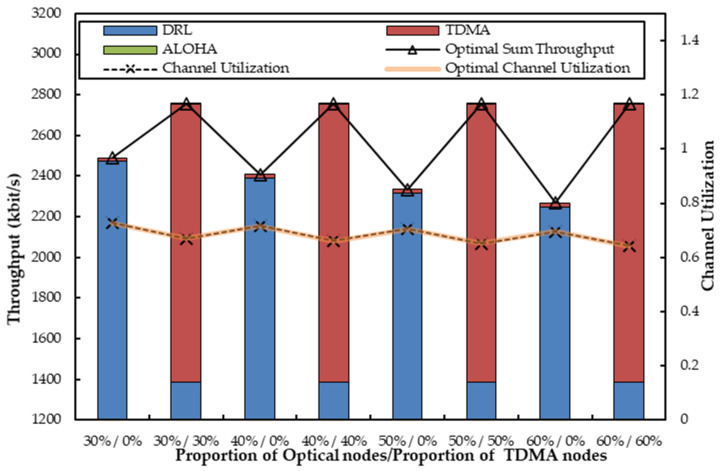
Network performances of different proportions of optical nodes and TDMA nodes.

For an extension, we consider a more complex scenario with multiple source nodes to observe how the network performance will be influenced by different ratios of the number of optical nodes to acoustic nodes. We employ a heterogeneous network with five agent source nodes and ten non-agent source nodes and seven channels (three optical channels and four acoustic channels), where all source nodes transmit packets to the common relay node. The number of optical nodes scales up with a step size of 10% of non-agent nodes, and we let the upper proportion of optical nodes be 60%. In other words, the numbers of optical nodes vary from 3 to 6 and the numbers of acoustic nodes vary from and 7 to 4. Besides, all nodes are classified into TDMA and ALOHA nodes. We set two combinations of TDMA and ALOHA nodes, considering each proportion of optical nodes. The proportions of TDMA nodes are set at 0% as the minimum and the current proportion of optical nodes as the maximum. That is, the minimum means that all optical nodes are ALOHA nodes, and all acoustic nodes are TDMA nodes. The maximum means that all optical nodes are TDMA nodes, and all acoustic nodes are ALOHA nodes. We no longer discuss what will happen if *R* or *q* changes since the related discussions have been stated in Section 6.2. Therefore, the number of time slots occupied by TDMA nodes in each optical channel *R* is 5 and the transmission probability *q* of ALOHA nodes is 0.1 as well. The corresponding simulation results are listed as follows.

(1.) *Short-term Sum Throughput:* Figure 11 exhibits the network throughput when non-agent nodes transmit data to the relay node employing acoustic and optical channels in different proportions, where the combinations of TDMA nodes and ALOHA nodes are also alterable. When all optical nodes are ALOHA nodes and all acoustic nodes are TDMA nodes, we notice that the throughput of TDMA nodes on acoustic channels is nearly the same because they transmit with a fixed number of time slots. Because only part of the agents can compensate for free optical channels, the remaining agents can compensate for unused acoustic channels. The throughput provided by agents acting as compensators on acoustic channels is also unchangeable. With a high-speed transmission capability, the optical channel’s throughput has a much greater impact on the sum throughput than the acoustic channel, so the sum throughput gradually decreases with the increasing number of ALOHA nodes. More ALOHA nodes mean more contention on the optical channel, resulting in a reduction in optical channel throughput.

On the contrary, when all optical nodes are TDMA nodes and all acoustic nodes are ALOHA nodes, the throughput of ALOHA nodes on acoustic channels is so extremely low that it is almost invisible in the figure because *q* is small, and an acoustic channel accommodates less transmission than an optical channel. Accordingly, the throughput provided by the agents to compensate for acoustic channels is also limited. Figure 11 also shows that the throughput of the optical TDMA nodes is unchangeable since they transmit packets through optical channels with a fixed number of time slots. The agents can assist in utilizing the remaining slots of the TDMA nodes on optical channels. Hence, the maximum throughput of the optical channels can always be achieved. As a result, the sum throughput remains nearly constant. Furthermore, we can easily see that in all cases, the total throughput can reach the near-optimal value discussed in Appendix A.2. Concurrently, a higher throughput can be obtained when all optical nodes are TDMA nodes and all acoustic nodes are ALOHA nodes.

(2.) *Channel Utilization:* Accordingly, Figure 11 illustrates the corresponding channel utilization. It also demonstrated that the simulated results are near optimal values because OA-DLMA always takes the optimal actions to optimize the channel utilization. In different scenarios, channel utilization follows a similar pattern to total throughput. Although channel utilization is higher when all optical nodes are ALOHA nodes and all acoustic nodes are TDMA nodes, there is little difference between the two scenarios. It is worth it to sacrifice a little channel utilization for higher throughput. That is why, in Section 6.3.3, the simulations are based on optical TDMA node and acoustic ALOHA node scenarios.

#### 6.3.3. OA-DLMA versus OA-CMAC/MC-DLMA Protocol

**Figure 12:**
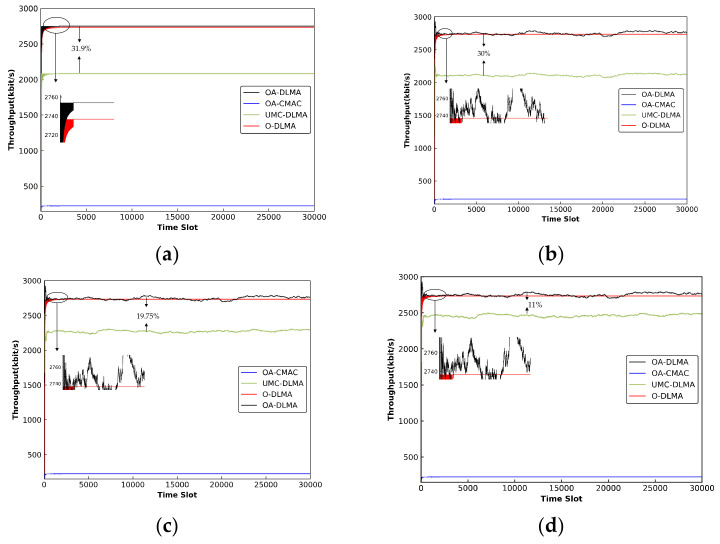
Sum throughput of the network with multiple source nodes using four MAC protocols (i.e., O-DLMA, OA-DLMA, OA-CMAC, and UMC-DLMA). The transmission probability *q* of ALOHA nodes in (**a**), (**b**), (**c**), and (**d**) is 0, 0.3, 0.5, and 0.7, respectively.

For an extension, we compare the throughput and channel utilization of our proposed OA-DLMA protocol with two typical MAC protocols, including the OA-CMAC protocol [39] and the underwater version of the MC-DLMA protocol [49], denoted as UMC-DLMA. The UMC-DLMA protocol has the same underwater configuration as the OA-DLMA and it can send packets via acoustic and optical channels. Furthermore, to demonstrate the benefit to each agent of compensating an optical or acoustic channel, we additionally compare our protocol with a benchmark protocol in which each agent only compensates for an optical channel (i.e., only transmits in the unused time slots of an optical channel), referred to as O-DLMA. We consider a scenario in which 15 source nodes attempt to communicate with one upper relay node through 3 optical channels and 4 acoustic channels, with TDMA nodes transmitting via optical channels and ALOHA nodes sending via acoustic channels. We enumerate four levels of network performance on the basis of different ALOHA node transmission probabilities to roundly finish the comparisons. That is, ALOHA nodes transmit with a transmission probability of 0, 0.3, 0.5, and 0.7, respectively.

(1.) *Short-term Sum Throughput:* Figure 12 exhibits the throughput of OA-DLMA, O-DLMA, OA-CMAC, and UMC-DLMA protocols with different values of *q* (*q* = 0, 0.3, 0.5, and 0.7). Regardless of *q* value, the throughputs of the three DRL-based protocols (OA-DLMA, O-DLMA, and UMC-DLMA) far outweigh those of the OA-CMAC protocol. The reason is that OA-CMAC protocol requests to send packets via acoustic and optical handshakes, which reduces the time of data packet sending. Furthermore, because the DRL algorithm is not adopted in it, unused time slots cannot be acquired and utilized effectively, which further degrades its throughput.

We can also see that OA-DLMA almost performs better than UMC-DLMA with different values of *q*. Although applying optimal configurations, UMC-DLMA can only achieve an optimal sum throughput of 2087.71 kb/s when *q* = 0, while in OA-DLMA 2754.43 kb/s. The reason for this is that, despite the fact that the UMC-DLMA protocol uses multi-channels to send packets, the UMC-DLMA nodes cannot most effectively acquire unused time slots in the high-capacity optical channels because the acoustic and optical channels are not distinguished in it, so the agents will randomly choose an underutilized channel for training and learning. On the contrary, our proposal discusses the transmitting ability difference between the acoustic and optical channels. Distinguishing rewards correspond to two different channels, which makes it beneficial to differentiate the specific actions on the acoustic channel or the optical channel. As a result, the agents will always prefer to compensate for the underutilized optical channels since the optical technology allows high speed and large data transmissions.

With an increase of *q*, the difference between OA-DLMA and UMC-DLMA becomes smaller. When *q* = 1, the two protocols have nearly the same throughput. The main reason for this is that a higher value of *q* increases the likelihood of the ALOHA node launching acoustic transmissions. Accordingly, the agent has less chance of acting as a compensator for sending packets in acoustic channels. Although there is no difference in reward values for successful optical channel transmissions and successful acoustic channel transmissions in UMC-DLMA, because fewer and fewer acoustic time slots are available, the agents can only use unoccupied time slots of TDMA nodes in optical channels. As a result, the difference between OA-DLMA and UMC-DLMA is almost negligible.

As shown in Figure 12, the throughput of O-DLMA is close to but lower than that of OA-DLMA. The main reason is that O-DLMA nodes can only compensate for free optical channels. Even if there are no free optical channels but multiple available acoustic channels, the agent cannot act as a compensator to send on acoustic channels. As a result, the transmission capacity of the free acoustic channel is wasted. OA-DLMA, on the other hand, allows each agent to compensate for an underutilized optical or acoustic channel. It can fully utilize the unused channels, resulting in higher throughput than O-DLMA. OA-DLMA has a maximum throughput of 2754.43 kb/s, while O-DLMA has a maximum throughput of nearly 2736.84 kb/s. The difference between O-DLMA and OA-DLMA is insignificant due to the lower transmission capacity of acoustic channels. With an increase of *q*, the difference between OA-DLMA and O-DLMA becomes much smaller because ALOHA nodes with a higher *q* transmit more packets on acoustic channels and agents have fewer chances to compensate for acoustic channels.

Even though agents only compensate for underutilized optical channels, O-DLMA has a much higher throughput than UMC-DLMA. The reason for this is that UMC-DLMA nodes cannot effectively compensate for unused time slots in high-capacity optical channels because they do not distinguish between acoustic and optical channels. As a result, the agents will select an underutilized channel at random, such as an optical or acoustic channel. On the other hand, O-DLMA nodes always compensate for underutilized high-capacity optical channels, resulting in higher throughput than UMC-DLMA.

**Figure 13:**
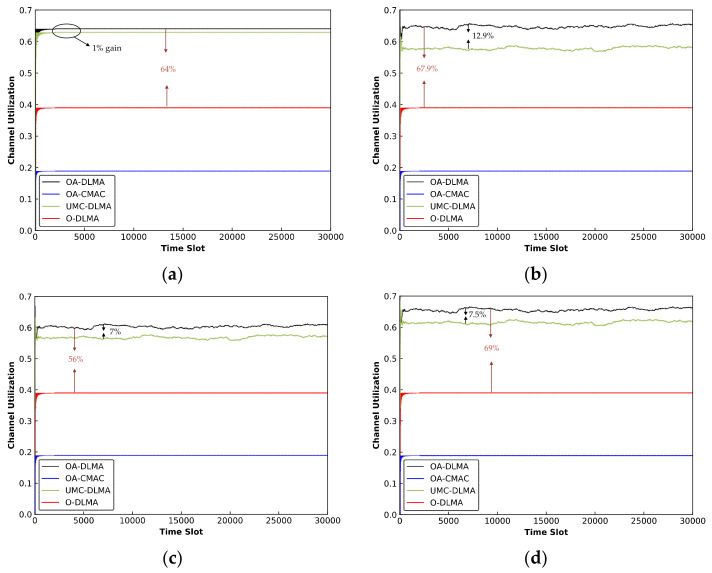
Channel Utilization of the network with multiple source nodes using four MAC protocols (i.e., O-DLMA, OA-DLMA, OA-CMAC, and UMC-DLMA). The transmission probability *q* of ALOHA nodes in (**a**), (**b**), (**c**), and (**d**) is 0, 0.3, 0.5, and 0.7, respectively.

All told, our proposal is advantageous in distinguishing between the specific actions on the acoustic and optical channels. Because of the high-speed data transmissions, the agents will always prefer to compensate for the underutilized optical channels. Furthermore, the agents can concurrently assist in making full use of acoustic and optical channels for packet transmissions. As a result, our protocol reflects better network performance than the three baseline protocols. The simulation results have demonstrated that our OA-DLMA protocol can achieve near-optimal sum throughput regardless of how non-agent nodes behave. Due to the participation of deep reinforcement learning, extra methods, such as the handshake mechanism, are not required in the OA-DLMA.

(2.) *Channel Utilization:* Figure 13 depicts the channel utilizations of OA-DLMA, O-DLMA, OA-CMAC, and UMC-DLMA protocols with different values of *q* (*q* = 0, 0.3, 0.5, and 0.7). Regardless of *q* value, the channel utilizations of the three DRL-based protocols (OA-DLMA, O-DLMA, and UMC-DLMA) far outperforms the OA-CMAC protocol. The reason is that OA-CMAC does not employ the DRL algorithm, and handshake mechanism further reduces the sending time of data packets. We can also see that OA-DLMA performs better than UMC-DLMA under different values of *q*. The reason is that the agents can learn the best policy to effectively acquire and utilize the unused time slots of different channels. Both OA-DLMA and UMC-DLMA outperform O-DLMA in terms of channel utilization because they can compensate for available optical and acoustic channels, whereas O-DLMA can only compensate for free optical channels. Even if there are no available optical channels but plenty of acoustic channels, the O-DLMA node cannot act as a compensator to send on acoustic channels. As a result, the free acoustic channel is squandered, leading to lower channel utilization.

## 7. Conclusions

In this paper, we proposed an optical and acoustic dual-channel deep-reinforcement learning multiple access protocol for heterogeneous underwater sensor networks, referred to as OA-DLMA. First, we present a heterogeneous underwater sensor network framework that integrates optical and acoustic transmissions, in which source nodes can deliver packets to the relay node via optical or acoustic channels using different slotted MAC protocols. Acoustic channels cooperating with optical channels always empower nodes to achieve stable long-distance communication and short-distance high-speed data transmission. In addition, deep reinforcement learning is introduced into the hybrid optical and acoustic dual-channel MAC design, which enables agent sensor nodes to acquire available time slots of underutilized channels (optical and acoustic) that are not used by non-agent nodes without knowing the transmission information in advance. Through an effective training mechanism, the agent nodes can be trained to capture and utilize the underutilized channels that are not entirely consumed by other nodes. Consequently, the goal of maximizing the throughput and channel utilization will be achieved by the continuous learning and training of the agents. Furthermore, in order to enhance the network performance, a distinct reward policy is set on the optical channel and the acoustic channel to differentiate the specific actions on the two different channels, with priority compensation encouraged for the optical channel due to its greater data transmission capability. More specifically, successful optical channel transmissions can earn a higher reward value than successful acoustic channel transmissions. Finally, we have derived the optimal short-term sum throughput and channel utilization analytically and conducted extensive simulations to evaluate the OA-DLMA protocol. The simulation results demonstrate that our protocol can adapt to the heterogeneous hybrid underwater optical and acoustic environment and perform with near-optimal performance. Compared with three benchmark protocols, our proposal can significantly improve the network performance in terms of sum network throughput and channel utilization.

In conclusion, the combination of the conventional optical and acoustic dual-channel MAC protocol and the DRL algorithm is validated for improving the network performance in the one-destination system. On the basis of this paper, we expect to investigate transmissions between multiple agent nodes and multiple destination nodes in future work. Furthermore, we have observed that the underwater channels are not always perfect, so transmissions may be lost in this scenario. As a result, future research will focus on how to improve network performance with imperfect underwater communication channels.

## Figures and Tables

**Table 1:** A comparison of different network models. (DR-DQN: Delayed-Reward Deep Q-Network, HetNets: Heterogeneous Networks.).

Research	Network	Communication Technique	Learning Algorithm	Channel Number	Main Contributions
Wang et al. [39]	UWSNs	Optical/Acoustic	N/A	Single Channel	Proposes an underwater optical and acoustic energy-efficient MAC protocol.
Park et al. [18]	UWSNs	Acoustic	RL	Single Channel	Proposes an underwater version of ALOHA-Q protocol with Q learning.
Geng et al. [24]	HetNets	Acoustic	DRL	Single Channel	Proposes an underwater DRL based MAC protocol and applies the protocol to both the synchronous and asynchronous time models.
Ye et al. [23]	HetNets	Acoustic	DRL	Single Channel	Provides a DR-DQN framework for proposing an DRL based MAC protocol for underwater HetNets.
Ye et al. [49]	HetNets	Radio	DRL	Multi-Channel	Proposes a DRL multi-channel MAC protocol for terrestrial HetNets.
Our study	HetNets	Optical/Acoustic	DRL	Dual Channel	Proposes a hybrid optical and acoustic DRL based MAC for underwater HetNets. To differentiate between the specific actions on the optical channel and the acoustic channel, a distinct reward policy is set for the two channels.

**Table 2:** OA-DLMA Hyper-parameters.

Hyper-Parameter	Value
The number of neurons per layer	64
Activation function	RELU
State history length *M*	32
Reward discount factor *γ*	0.9
Exploration probability *ε*	Decay from 1 to 0.01
Experience buffer capacity D	560
Random samples *N_E_*	32
Optimizer of DQN	RMSProp
Learning rate *α*	0.001
Update frequency *F* of target-net	480
Smoothing window size *N_w_*	1600

**Table 3:** OA-DLMA Parameters.

Parameter	Value
Acknowledgement time	0.1 s
Acoustic bit rate	10 kb/s
Optical bit rate	1 Mb/s
Maximum distance between source node and the relay node	30 m

## Data Availability

Not applicable.

## Abbreviations

The main abbreviations of this work are:

Abbreviation	Description
DNN	Deep Neural Network
DQL	Deep Q Learning
DQN	Deep Q-Network
DRL	Deep Reinforcement Learning
HOA-UWSNs	Hybrid Optical and Acoustic Underwater Wireless Sensor Networks
IoUT	Internet of Underwater Things
MAC	Media Access Control
OA-UWSN	Optical-Acoustic hybrid Underwater Wireless Sensor Network
RF	Radio Frequency
RL	Reinforcement Learning
ROVs	Remotely Operated Vehicles
SGD	Stochastic Gradient Descent
TWSNs	Terrestrial Wireless Sensor Networks
UASN	Underwater Acoustic Sensor Network
UAVs	Unmanned Aerial Vehicles
UWSNs	Underwater Wireless Sensor Networks
WSNs	Wireless Sensor Networks

## Appendix A. Derivations of Optimal Throughput and Channel Utilization

In the following, we introduce a model-aware node to replace the agent in our work since the agent is a DRL model-free node, which can finally sense the environment after learning and training. All the derivations in this paper are based on the work of Yu et al. [23,64].

### Appendix A.1. One Model-Aware Node Coexists with One TDMA Node and One ALOHA Node

We first consider the coexistence of a model-aware node with an acoustic TDMA node, and a model-aware node with an optical ALOHA node, respectively. For convenience of illustration, we analyze the throughput per unit time. Due to the different data transmission capabilities of different channels (optical and acoustic channels), the capacity in bit/s of the TDMA channel is set to *V*_1_ and the capacity in bit/s of the ALOHA channel is set to *V*_2_. When one model-aware node coexists with one TDMA node, the TDMA node transmits in *X* specific slots within each frame of *K* slots in a repetitive manner. In this case, the model-aware node is a TDMA-aware node that has full knowledge of the *X* slots used by the TDMA node. To maximize total throughput, the TDMA-aware node will transmit in all slots that the TDMA node is not using. Thus, the optimal total throughput is *V*_1_.

When one model-aware node coexists with one ALOHA node, the ALOHA node transmits with a fixed transmission probability of *q* in each time slot. The transmission probabilities of different time slots are independently and identically distributed. In a particular time slot, we let *p* be the transmission probability for the q-aware node and *f*(*p*) be the associated sum throughput of 1 model-aware node and (*N* − 1) q-ALOHA nodes. It is given as:

(A1) $$f\left(p\right)=V_{2}\cdot \left(N-1\right)\cdot q{\left(1-q\right)}^{N-2}\cdot \left(1-p\right)+V_{2}\cdot p{\left(1-q\right)}^{N-1}$$

The derivative of *f*(*p*) with respect to *p* is:

(A2) $$f^{\prime }\left(p\right)=V_{2}\cdot \left(1-N\right)\cdot q{\left(1-q\right)}^{N-2}+V_{2}\cdot {\left(1-q\right)}^{N-1}$$

As a result, we find that when $q>\frac{1}{N}$, $f^{\prime }\left(p\right)<0$; when $q<\frac{1}{N}$, $f^{\prime }\left(p\right)>0$. In other words, to optimize *f*(*p*), the optimal transmission probability *p* is as:

(A3) $$p^{*}=\left\{\begin{array}{ll} 0, & \;if\;q>\frac{1}{N}\\ 1, & \;if\;q\leq \frac{1}{N} \end{array}\right.$$

Thus, the corresponding optimal sum throughput $f\left(p^{*}\right)$ is given as:

(A4) $$f\left(p^{*}\right)=\left\{\begin{array}{ll} V_{2}\cdot (N-1)q{\left(1-q\right)}^{N-2}, & if\;q>\frac{1}{N}\\ V_{2}\cdot {\left(1-q\right)}^{N-1}, & if\;q\leq \frac{1}{N} \end{array}\right.$$

Considering the case where *N* = 2, we have learned from $f\left(p^{*}\right)$ that the ALOHA node does not transmit when *q* is no more than 0.5 and the agent will replace it. Besides, the ALOHA node transmits its data packets when *q* is greater than 0.5 and the agent will not transmit on the same channel. To further discuss the network performance when TDMA and ALOHA node coexist with model-aware node, we introduce some specific symbols. $N_{i}\left(i=1,2\right)$ denotes the numbers that acoustic transmission or optical transmission succeeds at the relay node during the whole measuring duration. *M* represents the overall frames during the simulation duration. We let $V_{1}=R_{1}$ denote the acoustic bit rate and $V_{2}=R_{2}$ denote the optical bit rate. $T_{i}\left(i=1,2\right)$ denotes the duration for sending acoustic data stream or optical data stream in one time slot, respectively.

#### Appendix A.1.1. Throughput with One Acoustic TDMA Node and One Optical ALOHA Node

First, we divide the sum throughput into two parts. The first part includes the throughput of the optical ALOHA node and the throughput that the model-aware node compensates for the optical channel. Another part includes the throughput of the acoustic TDMA node and the throughput that the model-aware node compensates for the acoustic channel. Because the optical channel transmits at high speeds, we focus primarily on its throughput.

According to Equation (15) of Section 6, the first part of the sum throughput is given by:

(A5) $${\mathrm{Throughput}}_{1}=\left\{\begin{array}{ll} \frac{R_{2}\cdot T_{2}\cdot \left(1-q\right)\cdot K\cdot M}{K\cdot M\cdot t_{slot}}=\frac{R_{2}\cdot T_{2}\cdot \left(1-q\right)}{t_{slot}}, & q\leq 0.5\\ \frac{R_{2}\cdot T_{2}\cdot q\cdot K\cdot M}{K\cdot M\cdot t_{slot}}=\frac{R_{2}\cdot T_{2}\cdot q}{t_{slot}}, & q>0.5 \end{array}\right.\;bit/s$$

The second part of the sum throughput is given by:

(A6) $${\mathrm{Throughput}}_{2}=\left\{\begin{array}{ll} \frac{R_{1}\cdot T_{1}\cdot \frac{X}{K}\cdot M\cdot K}{K\cdot M\cdot t_{slot}}=\frac{R_{1}\cdot T_{1}\cdot X}{K\cdot t_{slot}}, & q\leq 0.5\\ \frac{R_{1}\cdot T_{1}\cdot \frac{X}{K}\cdot M\cdot K+R_{1}\cdot T_{1}\cdot \frac{K-X}{K}\cdot M\cdot K}{K\cdot M\cdot t_{slot}}=\frac{R_{1}\cdot T_{1}}{t_{slot}}, & q>0.5 \end{array}\right.\;bit/s$$

The optimal sum throughput is calculated as the sum of Throughput_1_ and Throughput_2_:

(A7) $$\mathrm{Throughput}=\left\{\begin{array}{ll} \frac{R_{2}\cdot T_{2}\cdot \left(1-q\right)+R_{1}\cdot T_{1}\cdot \frac{X}{K}}{t_{slot}}, & q\leq 0.5\\ \frac{R_{2}\cdot T_{2}\cdot q+R_{1}\cdot T_{1}}{t_{slot}}, & q>0.5 \end{array}\right.\;bit/s$$

#### Appendix A.1.2. Throughput with One Acoustic ALOHA Node and One Optical TDMA Node

Then, we continue to divide the sum throughput into two parts. The first part includes the throughput of the optical TDMA node and the throughput that the model-aware node compensates for the optical channel. Another part includes the throughput of the acoustic ALOHA node and the throughput that the model-aware node compensates for the acoustic channel. The first part of the sum throughput is as:

(A8) $${\mathrm{Throughput}}_{1}=\frac{R_{2}\cdot T_{2}\cdot \frac{X}{K}\cdot M\cdot K+R_{2}\cdot T_{2}\cdot \frac{K-X}{K}\cdot M\cdot K}{K\cdot M\cdot t_{slot}}=\frac{R_{2}\cdot T_{2}}{t_{slot}}\;bit/s$$

The second part of the sum throughput is given by:

(A9) $${\mathrm{Throughput}}_{2}=\left\{\begin{array}{ll} \frac{R_{1}\cdot T_{1}\cdot \left(1-q\right)}{t_{slot}}, & q\leq 0.5\;and\;X=K\\ \frac{R_{1}\cdot T_{1}\cdot q}{t_{slot}}, & q\leq 0.5\;and\;X\neq K\;\\ \frac{R_{1}\cdot T_{1}\cdot q}{t_{slot}}, & q>0.5 \end{array}\right.\;bit/s$$

The optimal sum throughput is also calculated as the sum of Throughput_1_ and Throughput_2_:

(A10) $$\mathrm{Throughput}=\left\{\begin{array}{ll} \frac{R_{1}\cdot T_{1}\cdot \left(1-q\right)+R_{2}\cdot T_{2}}{t_{slot}}, & q\leq 0.5\;and\;X=K\\ \frac{R_{1}\cdot T_{1}\cdot q+R_{2}\cdot T_{2}}{t_{slot}}, & q\leq 0.5\;and\;X\neq K\;\\ \frac{R_{1}\cdot T_{1}\cdot q+R_{2}\cdot T_{2}}{t_{slot}}, & q>0.5 \end{array}\right.\;bit/s$$

#### Appendix A.1.3. Channel Utilization with One Acoustic TDMA Node and One Optical ALOHA Node

As illustrated in Equation (16) of Section 6, the channel utilization is given by:

(A11) $$\mathrm{Channel}\;\mathrm{Utilization}=\left\{\begin{array}{ll} \frac{\left[T_{2}\cdot \left(1-q\right)\cdot K+T_{1}\cdot \frac{X}{K}\cdot K\right]\cdot M}{K\cdot M\cdot C\cdot t_{slot}}=\frac{T_{2}\cdot \left(1-q\right)+T_{1}\cdot \frac{X}{K}}{t_{slot}\cdot C}, & q<0.5\\ \frac{T_{2}\cdot q\cdot K\cdot M+T_{1}\cdot \frac{X}{K}\cdot K\cdot M+T_{1}\cdot \frac{K-X}{K}\cdot K\cdot M}{K\cdot M\cdot C\cdot t_{slot}}=\frac{T_{2}\cdot q+T_{1}}{t_{slot}\cdot C}, & q>0.5 \end{array}\right.$$

#### Appendix A.1.4. Channel Utilization with One Acoustic ALOHA Node and One Optical TDMA Node

The channel utilization is given by:

(A12)
$$\mathrm{Channel}\;\mathrm{Utilization}=\left\{\begin{array}{ll} \frac{T_{2}+T_{1}\cdot \left(1-q\right)}{t_{slot}\cdot C}, & q\leq 0.5\;and\;X=K\\ \frac{T_{2}+T_{1}\cdot q}{t_{slot}\cdot C}, & q\leq 0.5\;and\;X\neq K\\ \frac{T_{2}+T_{1}\cdot q}{t_{slot}\cdot C}, & q>0.5 \end{array}\right.$$

### Appendix A.2. Y Model-Aware Nodes Coexist with Multiple Optical TDMA Nodes and Multiple Acoustic ALOHA Nodes

When multiple model-aware nodes coexist with multiple optical TDMA nodes and multiple acoustic q-ALOHA nodes, the optimal strategy is similar to the single model-aware node case. Model-aware nodes can still know the non-agent nodes’ propagation information of used slots. They can occupy the unused time slots that are not used by their adjacent non-agent nodes to maximize the network performance. We regard *Y* model-aware nodes as one integrated model-aware node. In each time slot, the integrated model-aware node decides whether the model-aware nodes should access the channel or not. If the integrated model-aware node finds a channel that can be accessed, it will choose a model-aware node out of all the model-aware nodes in a round-robin manner to access the available channel. Consider *Y* model-aware nodes coexisting with multiple TDMA nodes and multiple q-ALOHA nodes. We assume that there are *Z* optical channels and *W* acoustic channels. On a specific optical channel c_1_, all TDMA nodes transmit in *X* specific slots within each frame of *K* slots in a repetitive manner. On a specific acoustic channel c_2_, each q-ALOHA node transmits with a fixed transmission probability of *q* in each time slot. The optical channel c_1_ includes (Z_C1_-1) TDMA nodes and the acoustic channel c_2_ includes (W_C2_-1) ALOHA nodes. The other parameters in the following equations denote the same implications as in Appendix A.1. We continue to discuss the optimal sum throughput and channel utilization in this part.

#### Appendix A.2.1. Throughput When the Number of Model-Aware Nodes Is Less than That of Optical Channels

We still divide the sum throughput into two parts. The first part includes the throughput of optical channels, which includes the throughput of TDMA nodes and the model-aware nodes compensating for the optical channels. Another part includes the throughput of acoustic channels, which includes the throughput of ALOHA nodes and the model-aware nodes compensating for the acoustic channels. As shown in Equation (15) of Section 6, the first part of the sum throughput is given by:

(A13) $${\mathrm{Throughput}}_{1}=\left\{\begin{array}{l} \frac{Y\cdot R_{2}\cdot T_{2}+(Z-Y)\cdot R_{2}\cdot T_{2}\cdot \frac{X}{K}}{t_{slot}},X<K\\ \sum_{c_{1}=1}^{Z}\frac{R_{2}\cdot T_{2}}{t_{slot}},X=K \end{array}\right.\;bit/s$$

The second part of the sum throughput is given by:

(A14) $${\mathrm{Throughput}}_{2}=\left\{\begin{array}{l} \frac{\sum_{c_{2}=1}^{W}\left[\left(W_{c_{2}}-1\right)\cdot q\cdot R_{1}\cdot T_{1}\cdot {\left(1-q\right)}^{W_{c_{2}}-2}\right]}{t_{slot}},\;(q\leq \frac{1}{W_{c_{2}}}\;and\;X<K)\;or\;(q>\frac{1}{W_{c_{2}}})\\ \frac{\sum_{c_{2}=1}^{W}R_{1}\cdot T_{1}\cdot {\left(1-q\right)}^{W_{c_{2}}-1}}{t_{slot}},q\leq \frac{1}{W_{c_{2}}}\;and\;X=K \end{array}\right.\;bit/s$$

The optimal sum throughput is calculated as the total value of the two parts:

(A15) $$\mathrm{Throughput}=\left\{\begin{array}{ll} \frac{\sum_{c_{2}=1}^{W}\left[\left(W_{c_{2}}-1\right)\cdot q\cdot R_{1}\cdot T_{1}\cdot {\left(1-q\right)}^{W_{c_{2}}-2}\right]+R_{2}\cdot T_{2}\cdot \left[Y+\frac{X}{K}(Z-Y)\right]}{t_{slot}},(q\leq \frac{1}{W_{c_{2}}}\;and\;X<K)\;or\;(q>\frac{1}{W_{c_{2}}}\;and\;X<K) & \\ \frac{\sum_{c_{2}=1}^{W}\left[\left(W_{c_{2}}-1\right)\cdot q\cdot R_{1}\cdot T_{1}\cdot {\left(1-q\right)}^{W_{c_{2}}-2}\right]+\sum_{c_{1}=1}^{Z}\frac{R_{2}\cdot T_{2}}{t_{slot}}}{t_{slot}},\;q>\frac{1}{W_{c_{2}}}\;and\;X=K & bit/s\\ \frac{\sum_{c_{2}=1}^{W}R_{1}\cdot T_{1}\cdot {\left(1-q\right)}^{W_{c_{2}}-1}+\sum_{c_{1}=1}^{Z}\frac{R_{2}\cdot T_{2}}{t_{slot}}}{t_{slot}},q\leq \frac{1}{W_{c_{2}}}\;and\;X=K & \end{array}\right.$$

#### Appendix A.2.2. Throughput When the Number of Model-Aware Nodes Is Greater than the Number of Optical Channels but Not More Than the Total Number of Optical and Acoustic Channels

We still divide sum throughput into two parts. The first part includes the throughput of optical channels, which includes the throughput of TDMA nodes and the model-aware nodes compensating for the optical channels. Another part includes the throughput of acoustic channels, which includes the throughput of ALOHA nodes and the model-aware nodes compensating for the acoustic channels. As shown in Equation (15) of Section 6, the first part of the sum throughput is given by:

(A16) $${\mathrm{Throughput}}_{1}=\frac{Z\cdot R_{2}\cdot T_{2}}{t_{slot}}\;bit/s$$

The second part of the sum throughput is given by:

(A17) $${\mathrm{Throughput}}_{2}=\left\{\begin{array}{ll} \frac{\sum_{c_{2}=1}^{Y-Z}R_{1}\cdot T_{1}\cdot {\left(1-q\right)}^{W_{c2}-1}}{t_{slot}},q\leq \frac{1}{W_{c2}}\;and\;X\neq K & \\ \frac{\sum_{c_{2}=1}^{Y}R_{1}\cdot T_{1}\cdot {\left(1-q\right)}^{W_{c2}-1}}{t_{slot}},q\leq \frac{1}{W_{c2}}\;and\;X=K & bit/s\\ \frac{\sum_{c_{2}=1}^{Z}R_{1}\cdot T_{1}\cdot \left(W_{c2}-1\right)\cdot q\cdot {\left(1-q\right)}^{W_{c2}-2}}{t_{slot}},q>\frac{1}{W_{c2}} & \end{array}\right.$$

The optimal sum throughput is calculated as the total value of the two parts:

(A18) $$\mathrm{Throughput}=\left\{\begin{array}{ll} \frac{\sum_{c_{2}=1}^{Y-Z}R_{1}\cdot T_{1}\cdot {\left(1-q\right)}^{W_{c2}-1}+Z\cdot R_{2}\cdot T_{2}}{t_{slot}},q\leq \frac{1}{W^{c_{2}}}\;and\;X\neq K & \\ \frac{\sum_{c_{2}=1}^{Y}R_{1}\cdot T_{1}\cdot {\left(1-q\right)}^{W_{c2}-1}+Z\cdot R_{2}\cdot T_{2}}{t_{slot}},q\leq \frac{1}{W_{c2}}\;and\;X=K & bit/s\\ \frac{\sum_{c_{2}=1}^{Z}R_{1}\cdot T_{1}\cdot \left(W_{c2}-1\right)\cdot q\cdot {\left(1-q\right)}^{W_{c2}-2}+Z\cdot R_{2}\cdot T_{2}}{t_{slot}},q>\frac{1}{W_{c2}} & \end{array}\right.$$

##### Appendix A.2.3. Channel Utilization When the Number of Model-Aware Nodes Is Less than That of Optical Channels

The channel utilization is given by:

(A19)
$$\mathrm{Channel Utilization}=\left\{\begin{array}{l} \frac{Y\cdot T_{2}+(Z-Y)\cdot \frac{X}{K}\cdot T_{2}+\sum_{c_{2}}^{W}\left[\left(W_{c_{2}}-1\right)\cdot q\cdot T_{1}\cdot {\left(1-q\right)}^{W_{c_{2}}-2}\right]}{\left(Z+W\right)\cdot t_{slot}},(q\leq \frac{1}{W_{c_{2}}}\;and\;X<K)\;or\;(q>\frac{1}{W_{c_{2}}}\;and\;X<K)\\ \frac{\sum_{c_{2}}^{W}\left[\left(W_{c_{2}}-1\right)\cdot q\cdot T_{1}\cdot {\left(1-q\right)}^{W_{c_{2}}-2}\right]+\sum_{c_{1}=1}^{Z}T_{2}}{\left(Z+W\right)\cdot t_{slot}},q>\frac{1}{W_{c_{2}}}\;and\;X=K\\ \frac{\sum_{c_{2}}^{W}T_{1}\cdot {\left(1-q\right)}^{W_{c_{2}}-1}+\sum_{c_{1}=1}^{Z}T_{2}}{\left(Z+W\right)\cdot t_{slot}},q\leq \frac{1}{W_{c_{2}}}\;and\;X=K \end{array}\right.$$

##### Appendix A.2.4. Channel Utilization When the Number of Model-Aware Nodes is Greater than the Number of Optical Channels but Not More than the Total Number of Optical and Acoustic Channels

(A20)
$$\mathrm{Channel}\;\mathrm{Utilization}=\left\{\begin{array}{l} \frac{\sum_{c_{2}=1}^{Y-Z}T_{1}\cdot {\left(1-q\right)}^{W_{c2}-1}+\sum_{c_{2}=1}^{W-Z}T_{1}\cdot \left(W_{c2}-1\right)\cdot q\cdot {\left(1-q\right)}^{W_{c2}-2}+Z\cdot T_{2}}{\left(Z+W\right)\cdot t_{slot}},\;q\leq \frac{1}{W_{c2}}\;and\;X\neq K\\ \frac{\sum_{c_{2}=1}^{Y}T_{1}\cdot {\left(1-q\right)}^{W_{c2}-1}+\sum_{c_{2}=1}^{W-Y}T_{1}\cdot \left(W_{c2}-1\right)\cdot q\cdot {\left(1-q\right)}^{W_{c2}-2}+Z\cdot T_{2}}{\left(Z+W\right)\cdot t_{slot}},\;q\leq \frac{1}{W_{c2}}\;and\;X=K\\ \frac{\sum_{c_{2}=1}^{W}T_{1}\cdot \left(W_{c2}-1\right)\cdot q\cdot {\left(1-q\right)}^{W_{c2}-2}+Z\cdot T_{2}}{\left(Z+W\right)\cdot t_{slot}},\;q>\frac{1}{W_{c2}} \end{array}\right.$$
